# Design and Synthesis of Novel PRMT1 Inhibitors and Investigation of Their Effects on the Migration of Cancer Cell

**DOI:** 10.3389/fchem.2022.888727

**Published:** 2022-06-08

**Authors:** Caijiao Wang, Luyao Dong, Ziqi Zhao, Zeqing Zhang, Yutong Sun, Chonglong Li, Guoqing Li, Xuefu You, Xinyi Yang, Hao Wang, Wei Hong

**Affiliations:** ^1^ School of Chemistry and Chemical Engineering, North Minzu University, Yinchuan, China; ^2^ Beijing Key Laboratory of Antimicrobial Agents/Laboratory of Pharmacology, Institute of Medicinal Biotechnology, Chinese Academy of Medical Sciences and Peking Union Medical College, Beijing, China; ^3^ School of Pharmacy, Minzu University of China, Beijing, China; ^4^ School of Pharmacy, Ningxia Medical University, Yinchuan, China; ^5^ Key Laboratory of Ethnomedicine, Minzu University of China, Ministry of Education, Beijing, China; ^6^ Institute of National Security, Minzu University of China, Beijing, China; ^7^ Jingjinji National Center of Technology Innovation, Beijing, China

**Keywords:** PRMT, inhibitors, TGF-β, EMT, molecular docking

## Abstract

Protein arginine methyltransferase 1 (PRMT1) can catalyze the protein arginine methylation by transferring the methyl group from S-adenosyl-L-methionine (SAM) to the guanidyl nitrogen atom of protein arginine, which influences a variety of biological processes including epithelial–mesenchymal transition (EMT) and EMT-mediated mobility of cancer cells. The upregulation of PRMT1 is involved in a diverse range of cancer, such as lung cancer, and there is an urgent need to develop novel and potent PRMT1 inhibitors. In this article, a series of 2,5-substituted furan derivatives and 2,4-substituted thiazole derivatives were designed and synthesized by targeting at the substrate arginine-binding site on PRMT1, and 10 compounds demonstrated significant inhibitory effects against PRMT1. Among them, the most potent inhibitor, compound **1r** (WCJ-394), significantly affected the expression of PRMT1-related proteins in A549 cells and downregulated the expression of mesenchymal markers, by which WCJ-394 inhibited the TGF-β1-induced EMT in A549 cells and prevented the cancer cell migration. The current study demonstrated that WCJ-394 was a potent PRMT1 inhibitor, which could be used as the leading compound for further drug discovery.

## Introduction

Epithelial–mesenchymal transition (EMT) is an evolutionarily conserved developmental process that promotes the pathogenesis of many diseases including lung carcinoma and other lung injuries. During the process, epithelial cells lose their epithelial characteristics of apicobasal polarity and cell–cell junctions as well as acquire a behavioral phenotype that is commonly associated with mesenchymal cells (Wang et al., 2020). EMT-driven changes in plasticity by trans-differentiation into a mesenchymal phenotype endow lung epithelial cells or epithelial-derived tumor cells with metastatic properties, such as enhancing mobility, invasiveness, and resistance to apoptosis, which tend to suggest worse disease progression and higher mortality risk. Moreover, EMT also contributes to tumor cells with increased stem cell properties, chemotherapy resistance, and recurrence (Mittal, 2018). Given the role and the widely accepted hypothesis for EMT in the pathogenesis of lung cancers and other lung injuries, EMT is considered a hallmark of these diseases and targeting the EMT pathway represents an attractive therapeutic strategy (Salton et al., 2019). Transforming growth factor β (TGF-β) is a key regulatory cytokine to EMT. It drives the EMT program through multiple intracellular pathways, of which SMAD-dependent signaling is a very important one. In the TGF-β/SMAD signaling pathway, upon TGF-β activation, SMAD3 is phosphorylated and translocated into the nucleus with SMAD4 to downregulate or upregulate the transcription and expression of epithelial or mesenchymal phenotypic-associated genes, while SMAD7 inhibits the activation of SMAD3 (Katsuno et al., 2018; Wang et al., 2020). In view of the driving role of TGF-β on EMT, any effective regulator of the TGF-β/SMAD signaling pathway should be a potential EMT modulator, thereby affecting the migration and invasion of epithelial-derived cells (Wu et al., 2020).

Protein arginine methyltransferases (PRMTs) are a family of enzymes that catalyze the methylation of substrate protein arginine within biological systems. They can transfer the methyl group from S-adenosyl-L-methionine (SAM) to the guanidine nitrogen atom of protein arginine to form monomethylated arginine (MMA) and the cofactor S-adenosyl-L-homocysteine (SAH) (Blanc and Richard, 2017). Subsequently, MMA can be further methylated to generate the asymmetric dimethylarginine (ADMA) or symmetric dimethylarginine (SDMA) (Di Lorenzo and Bedford, 2011; Fulton et al., 2018; Zhang et al., 2021). Arginine methylation mediated by PRMTs is a common post-translational modification on both histone and non-histone proteins, which regulates various cellular biological procedures including cancer development (Lee et al., 2019). At present, a total of nine members of the PRMT family in the human body have been discovered, and according to the different methylation products, they can be divided into three types: type I PRMTs (PRMT1, 2, 3, 4, 6, and 8) catalyze the production of MMA and ADMA; type II PRMTs (PRMT5 and 9) catalyze the production of MMA and SDMA (Dominici et al., 2021); type III PRMT (PRMT7) only catalyze the production of MMA (Zurita-Lopez et al., 2012). Among them, PRMT1 is the most abundant and responsible for 90% of arginine methylation in mammalian cells (Lee et al., 2022). To date, enhanced PRMT1 expression has been well documented in a variety of cancers, including lung cancer, and is correlated with a poor prognosis of tumor development through its promotion on tumor cell growth, proliferation, invasion, and metastasis (Wang et al., 20211004; Wang et al., 2021; Yao et al., 2021; Yin et al., 2021; Hua et al., 2020; Repenning et al., 2021). PRMT1 is thus considered as a potential therapeutic target for lung cancer and metastasis. Furthermore, recent studies revealed that PRMT1 is an essential mediator of TGF-β/SMAD signaling and promotes the TGF-β-induced EMT through a mechanism of SMAD7 methylation. Therefore, PRMT1 inhibitors may exert their therapeutic effects in controlling tumor migration by inhibiting the EMT process, and changes in biomarkers and/or functions related to TGF-β/SMAD-mediated EMT can reflect the responses of tumor cells to PRMT1 inhibitors to a certain extent (Katsuno et al., 2018; Wei et al., 2019).

**FIGURE 1:**
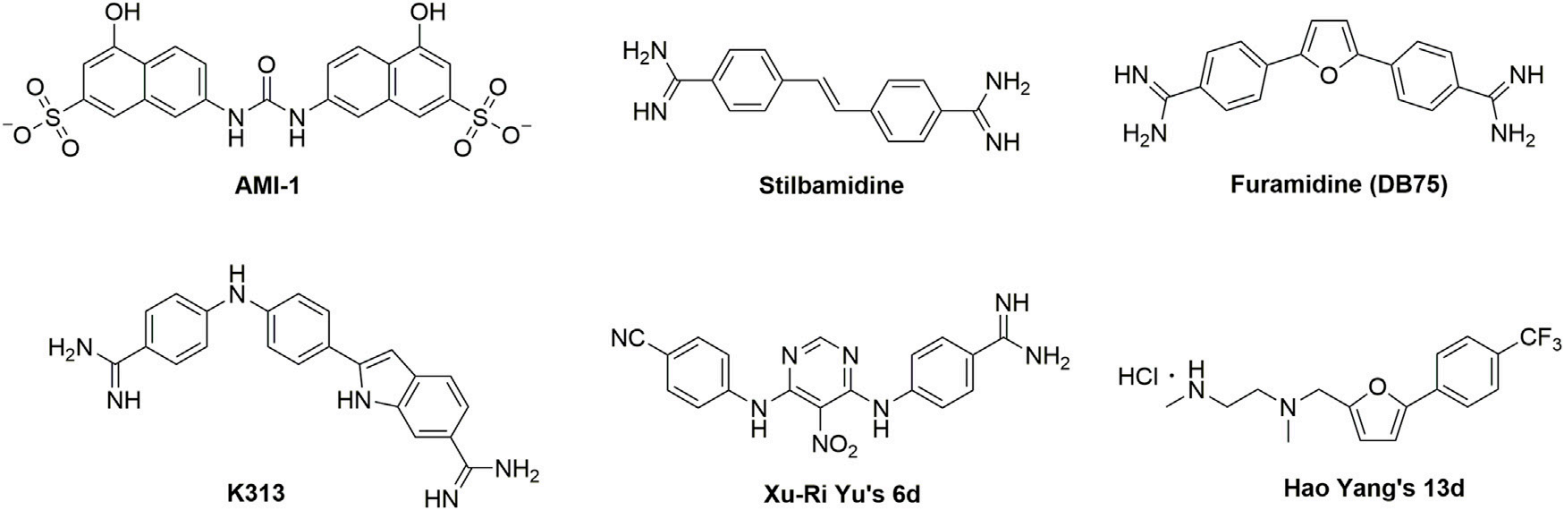
The chemical structures of the typical reported PRMT1 inhibitors.

Currently, a number of PRMT1 inhibitors have been reported (Figure 1). In 2004, AMI-1 was found as the first pan-PRMT inhibitor through high-throughput screening, which displayed selectivity to PRMTs against protein lysine methyltransferases (PKMTs) (Cheng et al., 2004). Later, a number of chemically symmetrical diamidine compounds as PRMT1 inhibitors with activities against cancer cells were reported, including stilbamidine (Spannhoff et al., 2007) and furamidine (DB75) (Yan et al., 2014). Subsequently, a more potent asymmetrical diamidine compound as PRMT1 inhibitor, K313 (Qian et al., 2021), was reported. Moreover, the compound Xu-Ri Yu’s 6d (Yu et al., 2015), which contains one amidine group, was also reported as a PRMT inhibitor. However, currently, there is no PRMT1 inhibitor in clinical usage, and there is an urgent need to discover more PRMT1 inhibitors with different clinical indications.

In our previous work (Yang et al., 2017), we discovered a furan ring-derived compound, Hao Yang’s 13d, with a potent PRMT1 inhibitory activity (IC_50_ value of 8.20 μM). Moreover, by using the molecular dynamic simulation, we assumed that the inhibitory activities could be further improved by introducing hydrogen donor groups on the benzene ring of compound Hao Yang’s 13d. Therefore, in the current work, a series of compounds bearing hydrogen donor groups (such as amide, hydrazide, amino, and N-hydroxyamidino and amidino) were designed and synthesized. Among them, compound **1r** (WCJ-394) was discovered to be a potent PRMT1 inhibitor, and the following studies showed that WCJ-394 significantly affected the expression of PRMT1-related proteins and inhibited the TGF-β1-induced EMT in A549 cells, which led to a significant inhibition on the cancer cells’ invasion and metastasis. Therefore, WCJ-394 could be an important leading compound for future PRMT1-guided drug discovery.

## Results and Discussion

### Chemistry

**SCHEME 1:**
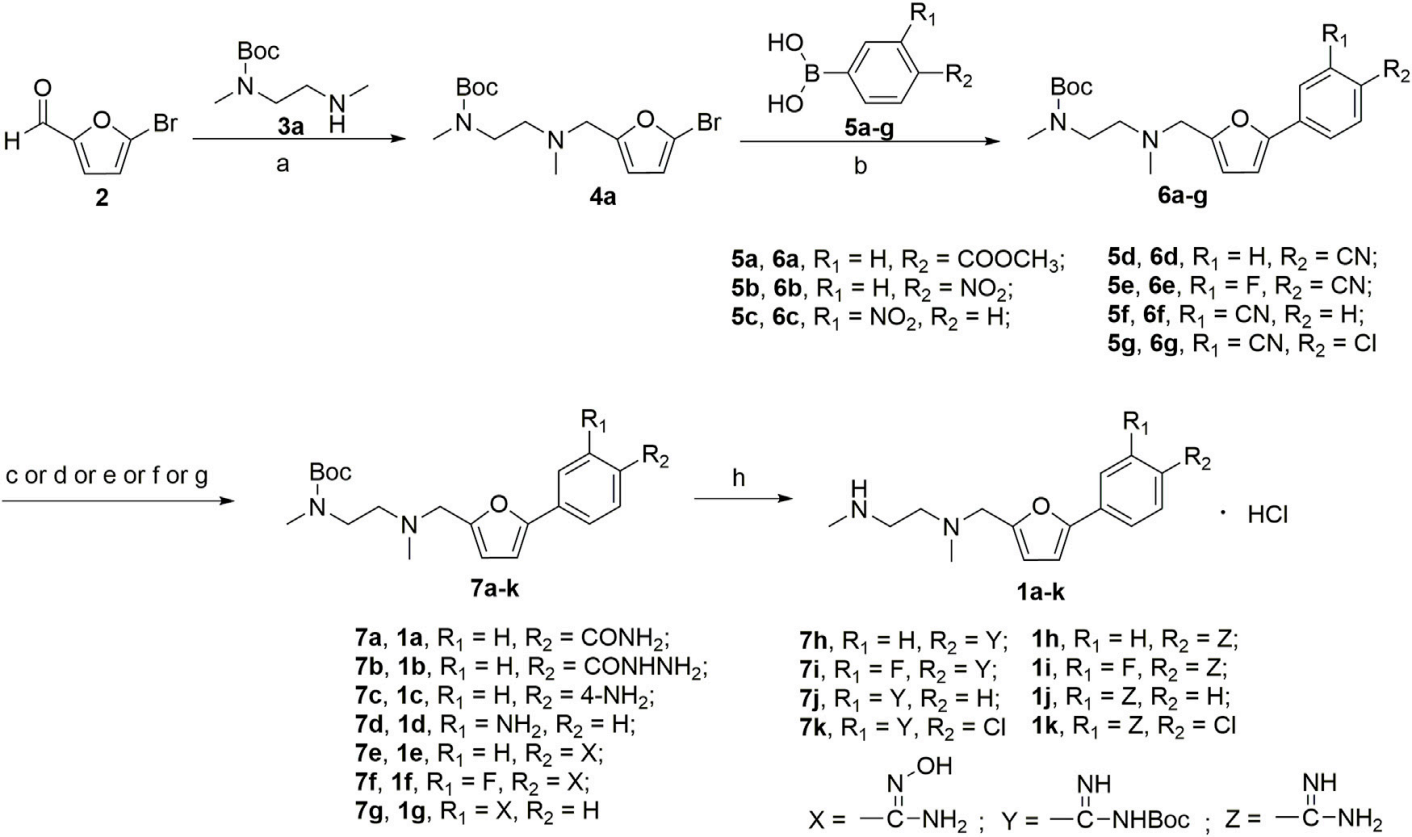
**(a)** NaBH(AcO)_3_, ClCH_2_CH_2_Cl; **(b)** 1,1′-di-*tert*-butylphosphinoferrocene palladium dichloride, K_2_CO_3_, toluene/EtOH/H_2_O; **(c)** EtONa, formamide, DMF; **(d)** NH_2_NH_2_, ethanol; **(e)** NH_2_NH_2_, Pd/C, isopropanol; **(f)** NHOH‧HCl, Et_3_N, dry EtOH; **(g)** LiN[Si(CH_3_)_3_]_2_, 2N HCl, dry THF, and then (Boc)_2_O, Et_3_N, CH_2_Cl_2_; **(h)** HCl/EtOH.

**SCHEME 2:**
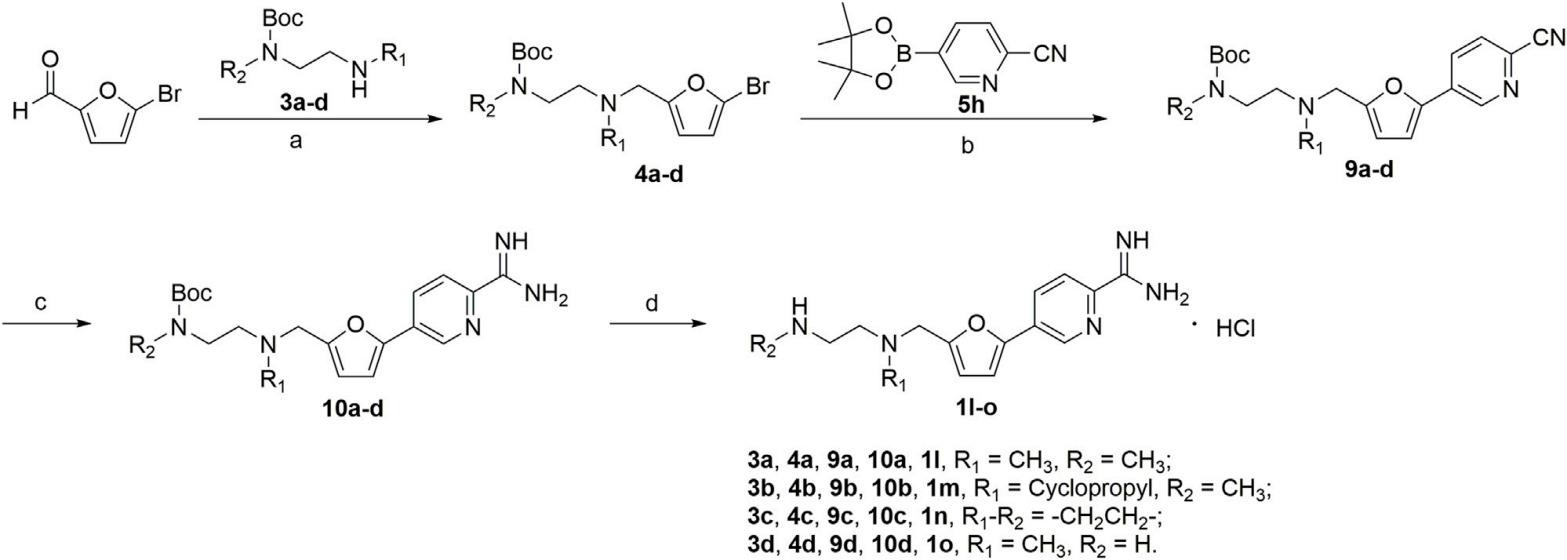
**(a)** NaBH(AcO)_3_, ClCH_2_CH_2_Cl; **(b)** 1,1′-di-*tert*-butylphosphinoferrocene palladium dichloride, K_2_CO_3_, toluene/EtOH/H_2_O; **(c)** CH_3_ONa, NH_4_Cl, CH_3_OH; **(d)** HCl/EtOH.

A series of 2,5-substituted furan derivatives **1a–o** were synthesized, as illustrated in Schemes 1, 2. In Scheme 1, the commercially available 5-bromofurfural was treated with ethylenediamine compound **3a** by reductive amination using sodium triacetoxyborohydride (STAB) in 1,2-dichloroethane to generate compound **4a** in an yield of 81%. Compound **4a** was reacted with different substituted phenylboric acids **5a–g** in the presence of catalyst 1,1′-di-tert-butylphosphinoferrocene palladium dichloride and base potassium carbonate in a mixed solvent (toluene/water/ethanol = 2/1/1) to generate compounds **6a–g** in moderate yields (70%–85%). And then, compound **6a–g** were converted to the precursor compound **7a–g** by the required conditions: compound **6a** was treated with sodium ethoxide and formamide to give amide compound **7a** in a yield of 72%; compound **6a** was reacted with hydrazine hydrate in ethanol to generate the hydrazide compound **7b** in a yield of 61%; compounds **6b,c** were converted to amino substituted compounds **7b,c** using hydrazine hydrate and Pd/C by catalytic transfer hydrogenation in a moderate yield (67%–79%); compounds **6d–f** were treated with hydroxylamine hydrochloride and triethylamine to give N-hydroxyamidino compounds **7e–g** in moderated yields (54%–63%); compounds **6d–g** were reacted with lithium bis(trimethylsilyl)amide and subsequently protection reaction to form protected amidino compounds **7h–k** in a yield of 32%–92%. Finally, the target compounds **1a–k** were obtained by deprotection of compounds **7a–k** with the saturated hydrochloric acid ethanol solution in good yields (80%–90%).

The synthesis of amidino derivatives **1l-o** is depicted in Scheme 2. The commercially available 5-bromofurfural was, respectively, treated with compounds **3a–d** by reductive amination using sodium triacetoxyborohydride (STAB) in 1,2-dichloroethane to generate compounds **4a–d** in moderate yields (70%–85%). And then, using potassium carbonate as a base and 1,1′-di-tert-butylphosphinoferrocene palladium dichloride as a catalyst, compounds **4a–d** were reacted with 2-cyanopyridine-5-borate ester **5h** in a mixed solvent (toluene/water/ethanol = 2/1/1) by the Suzuki reaction to generate compounds **9a–d** in low yields (50%–60%). Subsequently, similar to the procedure in Scheme 1, compounds **9a–d** were treated with the required reagents by the nucleophilic addition reaction to generate compounds **10a–d** in moderate yields (50%–80%), which were deprotected with the saturated hydrochloric acid ethanol solution to afford the target compounds **1l–o** in moderate yields (75%–85%).

**SCHEME 3:**
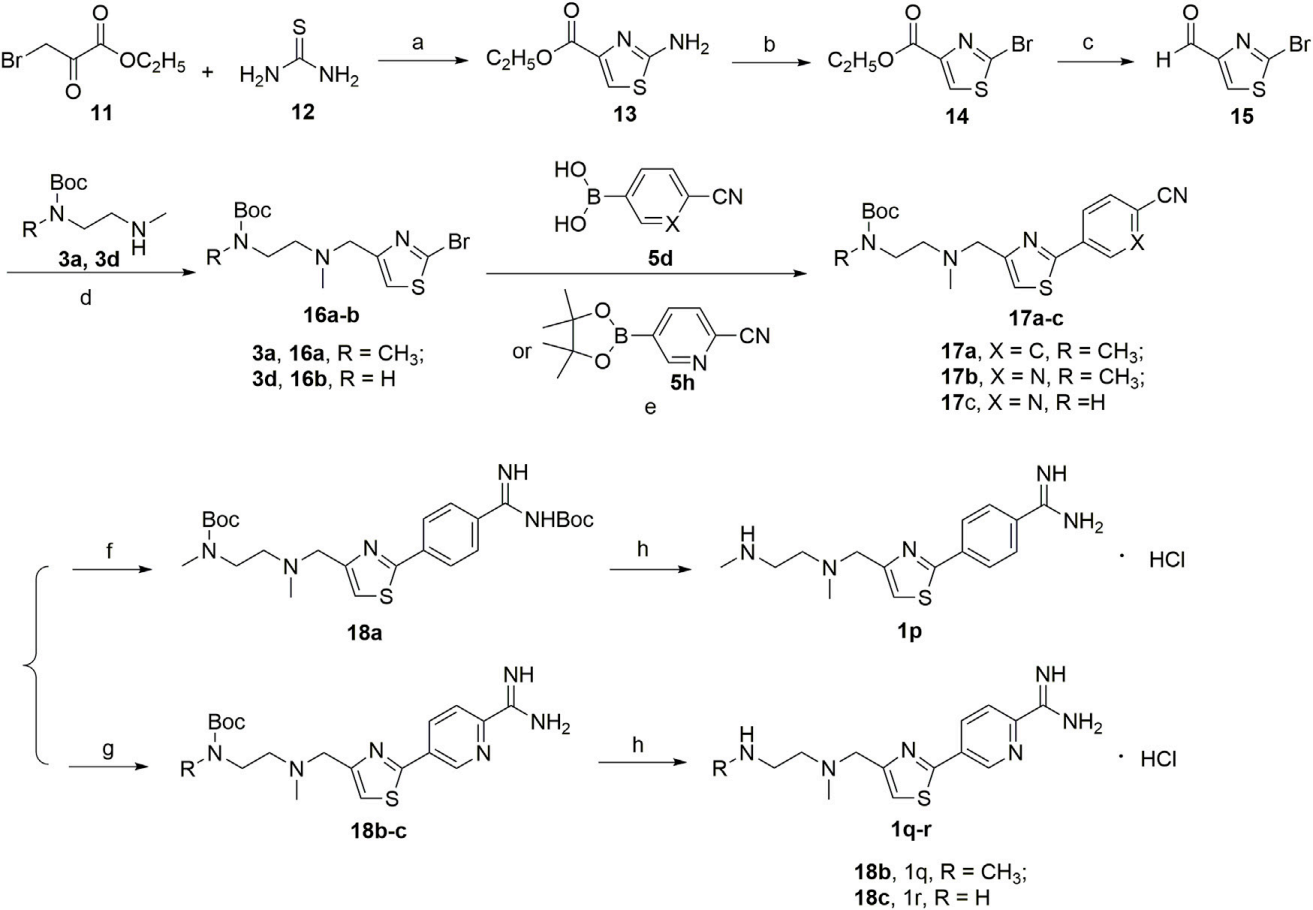
**(a)** EtOH; **(b)** CuBr_2_, C_4_H_9_NO_2_, CH_3_CN; **(c)** NaBH_4_, EtOH; Dess-Martin reagent, dry THF; **(d)** NaBH(AcO)_3_, ClCH_2_CH_2_Cl; **(e)** 1,1′-di-*tert*-butylphosphinoferrocene palladium dichloride, K_2_CO_3_, toluene/EtOH/H_2_O; **(f)** LiN[Si(CH_3_)_3_]_2_, 2N HCl, dry THF, and then (Boc)_2_O, Et_3_N, CH_2_Cl_2_; **(g)** CH_3_ONa, NH_4_Cl, CH_3_OH; **(h)** HCl/EtOH.

A series of 2,4-substituted thiazole derivatives **1p–r** were synthesized, as illustrated in Scheme 3. The commercially available 3-bromopyruvate ethyl was reacted with thiourea by cyclization reaction in absolute ethanol to generate 2-amino thiazole compound **13** in a yield of 93%. And then, compound **13** was treated with *tert*-butyl nitrite and copper dibromide in acetonitrile by diazotization and bromination reaction to give 2-bromo thiazole compound **14** in a low yield of 30%. Meanwhile, compound **14** was converted to aldehyde compound **15** sequentially by reductive reaction in the presence of sodium borohydride and oxidation reaction in the presence of the Dess–Martin reagent in a moderate yield of 72% over two steps. Similar to the procedure in Schemes 1, 2, compound **15** was, respectively, reacted with side chain **3a** or **3d** by reductive amination to generated compounds **16a,b** in moderate yields (55%–75%), which were treated with boronic acid **5d** or borate ester **5h** by the Suzuki reaction to generate compounds **17a–c** in moderate yields (55%–75%). Compounds **17a–c** were treated in the required conditions by the nucleophilic addition reaction to generate compounds **18a–c** in low yields (30%–70%), which were deprotected with the saturated hydrochloric acid ethanol solution to afford the target compounds **1p–r** in good yields (85%–90%).

### *In Vitro* PRMT1 Inhibition Assay

A series of 2,5-substituted furan and 2,4-substituted thiazole derivatives were synthesized and screened for their inhibitory effects on PRMT1 by using the radioactive PRMT1 methylation inhibition assay, which measured the amount of methyl groups that transferred from [^3^H]-SAM to a biotinylated histone H4 peptide [ac-SGRGKGGKGLGKGGAKRHRKVGGK(Biotin)] (Tables 1–3). In the assay, SAH and DB75 were used as the positive controls.

**TABLE 1:** Inhibitory activities of 2,5-substituted furan derivatives 1a–k against PRMT1.

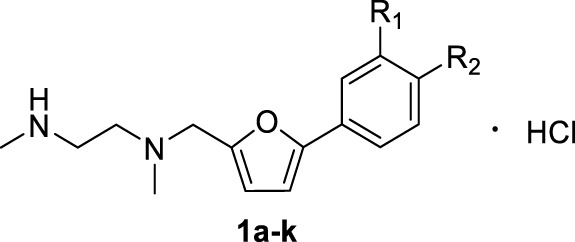					
**Cpd no.**	**Cpd ID.**	**R_1_**	R_**2**_	**% Inhibition of PRMT1 (at 10 μM)^a^**	**IC_50_ (μM)**
—	SAH	—	—	—	0.55 ± 0.07
—	DB75	—	—	98.13 ± 0.27	0.31 ± 0.04
**1a**	ZZQ-16	H	-CONH_2_	10.61 ± 2.24	—
**1b**	ZZQ-82	H	-CONHNH_2_	48.00 ± 0.02	—
**1c**	ZZQ-55	H	-NH_2_	50.00 ± 0.18	21.42 ± 12.76
**1d**	ZZQ-52	-NH_2_	H	35.99 ± 0.09	—
**1e**	ZZQ-102	H	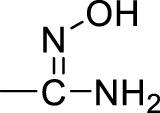	32.00 ± 0.00	—
**1f**	ZZQ-127	F	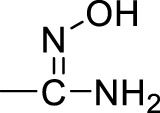	35.97 ± 0.20	—
**1g**	ZZQ-100	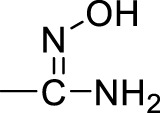	H	39.98 ± 0.19	—
**1h**	SYT-302	H	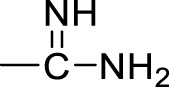	74.54 ± 0.16	2.66 ± 0.01
**1i**	SYT-298	F	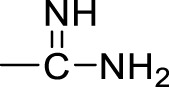	68.00 ± 0.02	4.42 ± 0.63
**1j**	SYT-290	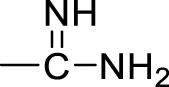	H	76.82 ± 1.19	2.06 ± 0.60
**1k**	SYT309	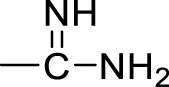	Cl	76.50 ± 0.01	2.50 ± 0.91

a Initial inhibition rate was tested at 10 μM for all compounds, and only those with >50% inhibition effects were selected for IC_50_ measurements.

**TABLE 2:** Inhibitory activities of 2,5-substituted furan derivatives 1l–o against PRMT1.

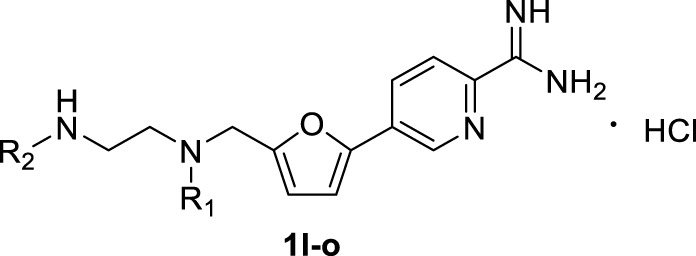					
**Cpd no.**	**Cpd ID.**	**R_1_**	**R_2_**	**% Inhibition of PRMT1 (at 10 μM)**	**IC_50_ (μM)**
—	SAH	—	—	—	0.55 ± 0.07
—	DB75	—	—	98.13 ± 0.27	0.31 ± 0.04
1l	WCJ-237	-CH_3_	-CH_3_	92.12 ± 0.28	1.29 ± 0.12
1m	WCJ-163	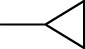	-CH_3_	48.00 ± 0.02	10.78 ± 1.30
1n	WCJ-77		-CH_2_CH_2_	73.51 ± 0.06	4.68 ± 0.26
1o	WCJ-173	-CH_3_	H	89.02 ± 0.05	2.41 ± 0.18

**TABLE 3:** Inhibitory activities of 2,4-substituted thiazole derivatives 1p–r against PRMT1.

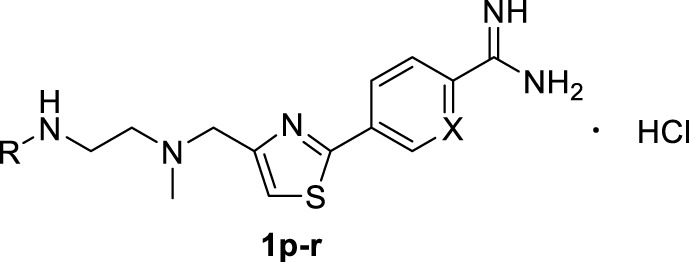					
**Cpd no.**	**Cpd ID.**	**X**	**R**	**% Inhibition of PRMT1 (at 10 μM)**	**IC_50_ (μM)**
—	SAH	—	—	—	0.55 ± 0.07
—	DB75	—	—	98.13 ± 0.27	0.31 ± 0.04
1p	WCJ-141	C	-CH_3_	87.00 ± 0.00	1.49 ± 0.47
1q	WCJ-172	N	-CH_3_	75.71 ± 0.82	4.14 ± 0.34
1r	WCJ-394	N	H	98.50 ± 0.04	1.21 ± 0.11

Initially, we designed and synthesized a series of molecules (**1a–k**), as shown in Table 1, which contained substituted phenyl on 5-furan and ethylenediamine side chain on 2-furan. The chemical modification of these compounds is mainly concentrated on the substituted benzene ring, such as amide, hydrazide, amino, and N-hydroxyamidino and amidino groups. Compared with compounds **1a–d**, it was found that the inhibitory rate of 4-amino-substituted compound (**1c**) was stronger than those of amide, hydrazide, or 3-amino-substituted compounds (**1a**, **1b**, and **1d**) at 10 μM against PRMT1 and was selected for the IC_50_ determination (21.42 ± 12.76 μM). It was shown that the introduction of the hydrogen bond donor group on the 4-phenyl group could improve the inhibitory activity slightly. However, it was noticed that introducing the N-hydroxyamidino group (the hydrogen bond donor group as well) on the benzene ring (compounds **1e**, **1f**, and **1g**) could not increase the inhibitory activity, whose inhibition rates were only 32%–40% at 10 μM against PRMT1. Subsequently, four compounds (**1h**, **1i**, **1j**, and **1k**), containing 4-amidino, 3-F-4-amidino, 3-amidino, and 3-amidino-4-Cl group, respectively, showed strong inhibitory effects (68%–77%) at 10 μM against PRMT1 and were selected for the IC_50_ determinations, which were 2.66 ± 0.01, 4.42 ± 0.63, 2.06 ± 0.60, and 2.50 ± 0.91 μM, respectively. Although the IC_50_s of these compounds are weaker than those of SAH and DB75, we found that introducing the hydrogen donor group, amidino on the benzene ring, could increase the inhibitory activity greatly (Table 1), which could be explained by the fact that the amidino group may mimic the guanidino group of substrate arginine.

In the second step, we assumed that the introduction of “N” into the benzene ring could increase the interaction between the compound and PRMT1; the benzene ring was transformed to the pyridine ring and different amino side chains were introduced to gain a series of molecules (**1l–o**), as shown in Table 2. Among them, it was found that four compounds (**1l, 1m, 1n, and 1o**) showed strong inhibitory rates (48%–92%) at 10 μM against PRMT1, and IC_50_s were determined as 1.29 ± 0.12, 10.78 ± 1.30, 4.68 ± 0.26, and 2.41 ± 0.18 μM, respectively. Introducing the pyridine ring on the 5-furan could improve the inhibitory activity slightly by comparing compounds **1l** and **1h–k**. However, compounds **1m** and **1n** bearing the cyclopropyl group and the piperazine group on the R1 position, respectively, have the weaker inhibitory activity than **1l**, probably because of the steric hindrance on the R1 position. Moreover, introducing the primary amine on the terminal amino group (**1o**) could have a similar inhibitory activity with **1l**. The IC_50_ of compound **1l** is only about two times weaker than SAH and four times weaker than DB75 (Table 2). So far, we found that introducing the pyridine bearing the amidino group on the 5-furan could perform relatively good inhibitory activities.

In the third step, in order to investigate the effects of other cores such as thiazole on the inhibitory activity of PRMT1, we designed and synthesized a series of compounds (**1p–r**, Table 3) based on the structure of compound **1l**. These three compounds showed relatively good inhibitory activities, and their inhibitory rates were higher than 75% at a concentration of 10 μM, and especially compounds **1p** and **1r** exhibited 1.49 ± 0.47 and 1.21 ± 0.11 μM IC_50_s, respectively, against PRMT1. Among all of the above compounds, it should be noted that compounds **1p** and **1r** have similar inhibitory activities and their IC_50_s are about two times weaker than SAH and three times weaker than DB75 (Table 3). In conclusion, among all the compounds, two most potent ones (**1l** and **1r**) were discovered with almost identical inhibitory activities, and **1r** was selected as the representative compound for the following study.

The selectivity profiles of compound **1r** against PRMT1 and other type I PRMTs (PRMT3, 4, 6, and 8) were investigated, and the IC_50_s were measured, as illustrated in Table 4. It can be observed that compound **1r** exhibited similar inhibitory activities against PRMT4 and PRMT8, which are stronger than those against PRMT3 and 6.

**TABLE 4:** Inhibitory activities (IC_50_, μM) of compound WCJ-394 against type I PRMTs.

Cpd ID.	PRMT3	PRMT4	PRMT6	PRMT8
WCJ-394	57.59 ± 0.62	1.64 ± 0.17	29.70 ± 2.03	5.71 ± 0.05

### The Alteration of Compound 1r (WCJ-394) on PRMT Patterns in A549 Cells

**FIGURE 2:**
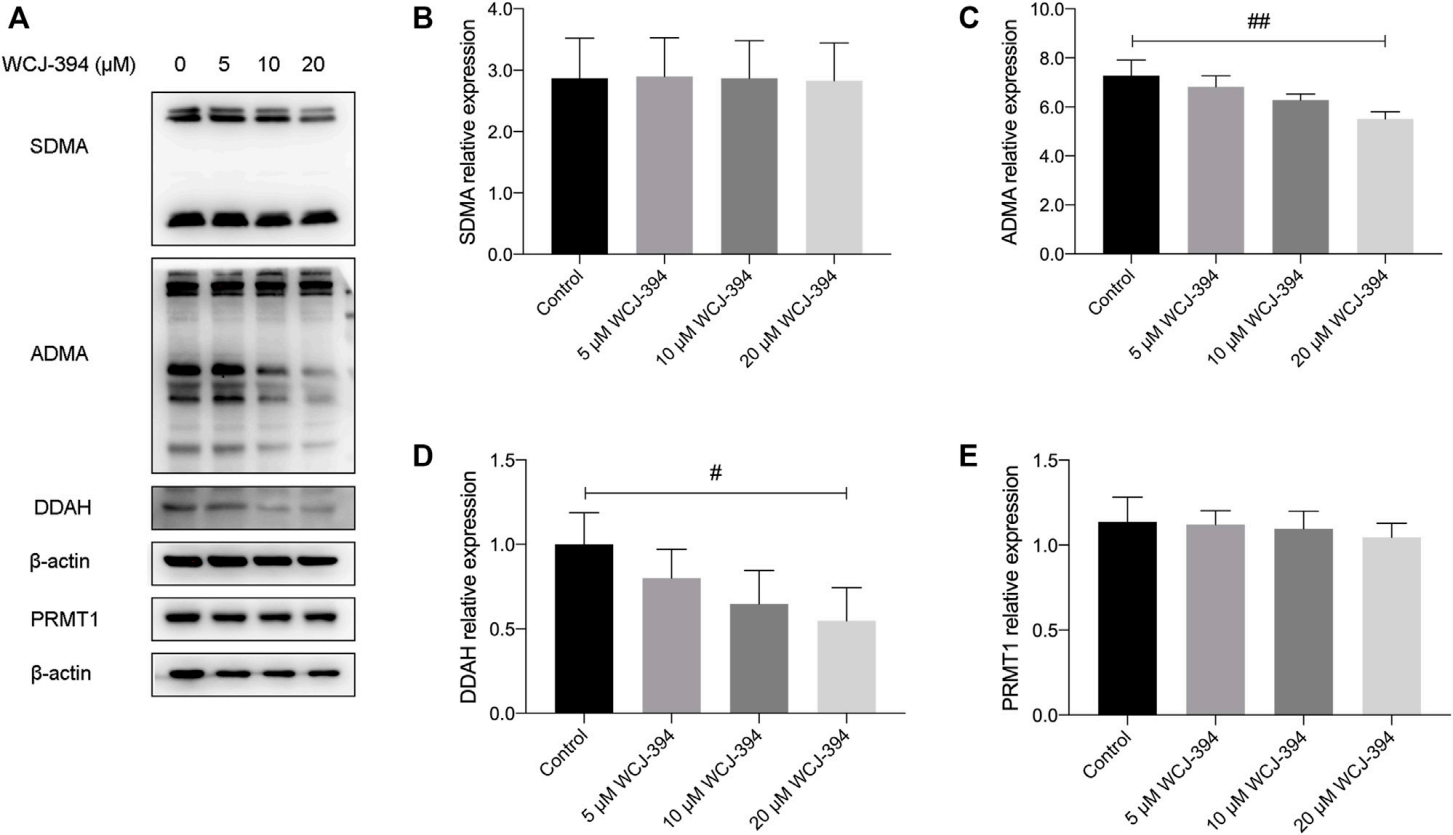
Effect of WCJ-394 on PRMT-related proteins in A549 cells. **(A)** Western blot result of symmetrical dimethylarginine (SDMA), asymmetrical dimethylarginine (ADMA), dimethylarginine dimethylaminohydrolase (DDAH), and protein arginine methyltransferase 1 (PRMT1) proteins in A549 cells treated with WCJ-394 (5, 10, 20 μΜ). **(B–E)** Expression levels of SDMA, ADMA, DDAH, and PRMT1 relative to the internal control β-actin. ^#^ *p* < 0.05, ^##^ *p* < 0.01 vs. the control group.

Western blotting was performed to confirm that WCJ-394 altered the arginine methylation patterns of PRMT1 in A549 cells. As shown in Figure 2, after the treatment of WCJ-394 in A549 cells for 48 h, the expression of PRMT1 was not significantly affected, and the expression level of ADMA, which is mainly produced by type I PRMTs, was significantly reduced in a concentration-dependent manner. In addition, dimethylarginine dimethylaminohydrolase (DDAH), which can metabolize more than 90% of ADMA (Chen et al., 2009), was also significantly decreased in a concentration-dependent manner. Taken together, the decrease of ADMA was because of the inhibition of PRMT1 by WCJ-394, rather than being metabolized by DDAH or decreasing the expression of PRMT1, which suggested that WCJ-394 directly influenced the PRMT–ADMA pathway. Unlike ADMA, the expression of SDMA, which was catalyzed by type II PRMTs, was not affected by WCJ-394. In conclusion, it was confirmed that WCJ-394 was a PRMT1 inhibitor and had significant effects on the PRMT pathway at cellular levels.

### Inhibition of Compound 1r (WCJ-394) on TGF-β Signaling

**FIGURE 3:**
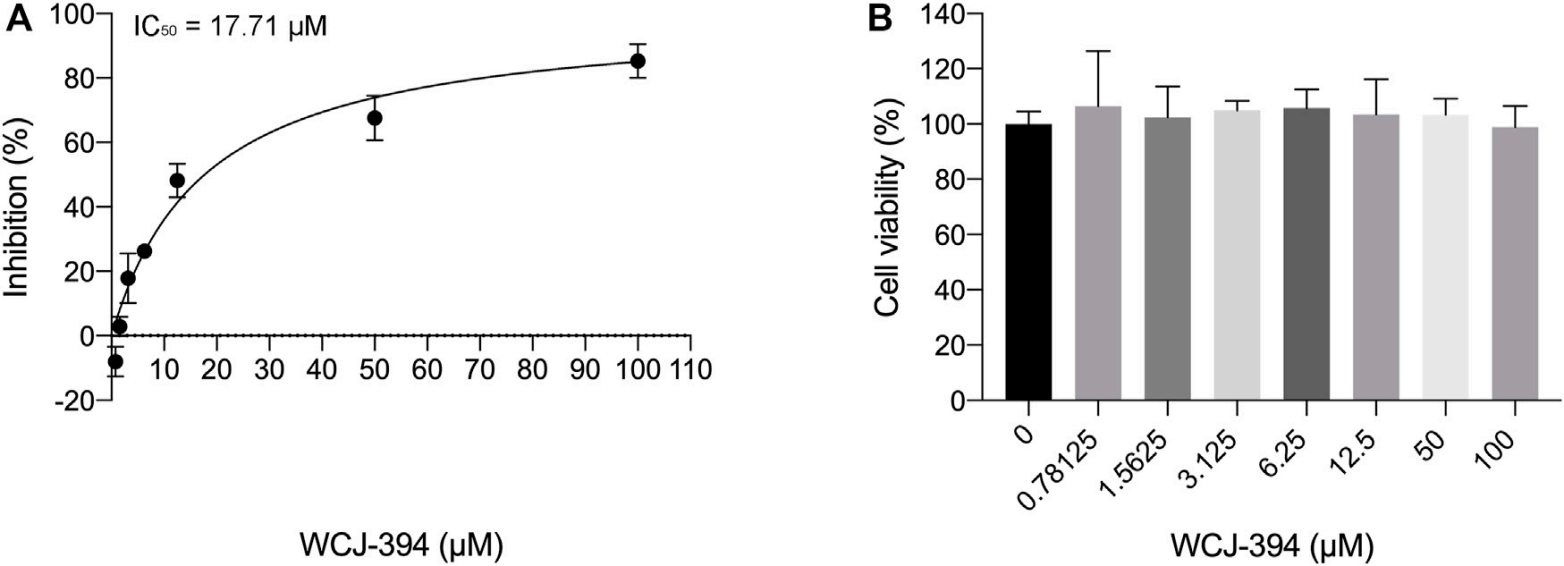
The inhibitory effect of WCJ-394 on the TGF-β signaling pathway. **(A)** Inhibition of WCJ-394 on the TGF-β signaling, IC_50_ = 17.71 μΜ. **(B)** Effect of WCJ-394 on MLEC cell viability in the same condition.

It was reported that PRMT1 may activate the TGF-β/SMAD3 signaling pathway (Katsuno et al., 2018; Wei et al., 2019); therefore, the ability of WCJ-394 to inhibit TGF-β signaling was measured by using the mink lung epithelial cell (MLEC), which was stably transfected with a TGF-β-responsive plasminogen activator inhibitor-1 promoter-luciferase construct (Abe et al., 1994). On the addition of TGF-β, a dose-dependent increase in luciferase activity can be induced, so that the inhibitory activity of compounds on TGF-β signaling pathway can be evaluated (Wu et al., 2020). As shown in Figure 3, WCJ-394 could significantly inhibit the TGF-β signaling with an IC_50_ of 17.71 μΜ, without showing significant cytotoxicity in a concentration up to 100 μΜ under the same condition.

### The Effects of Compound 1r (WCJ-394) on TGF-β-Induced EMT in A549 Cells

**FIGURE 4:**
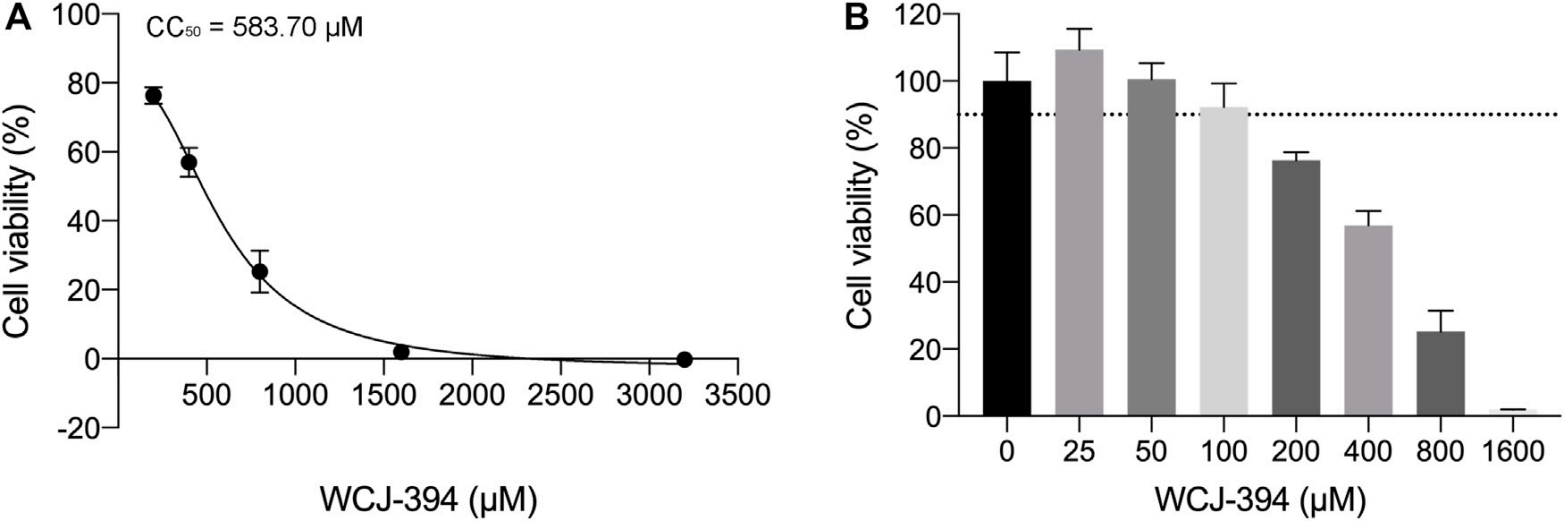
Potential cytotoxic effect of WCJ-394 on A549 cells. **(A)** Cell viability of A549 treated with WCJ-394, CC_50_ = 583.70 μΜ. **(B)** Effect of WCJ-394 on the A549 cell viability.

To determine the effects of WCJ-394 on the inhibition of TGF-β1-induced epithelial–mesenchymal transition (EMT) in A549 cells, the characterized proteins were detected using the Western blotting assay. Initially, the potential cytotoxic effect of WCJ-394 was examined through the CCK-8 cell viability assay and the results (Figure 4) suggested that WCJ-394 had an IC_50_ of 583.70 μM in A549 cells treated for 48 h and did not result in any cytotoxicity in this cell line at the concentrations of 0–50 μM.

**FIGURE 5:**
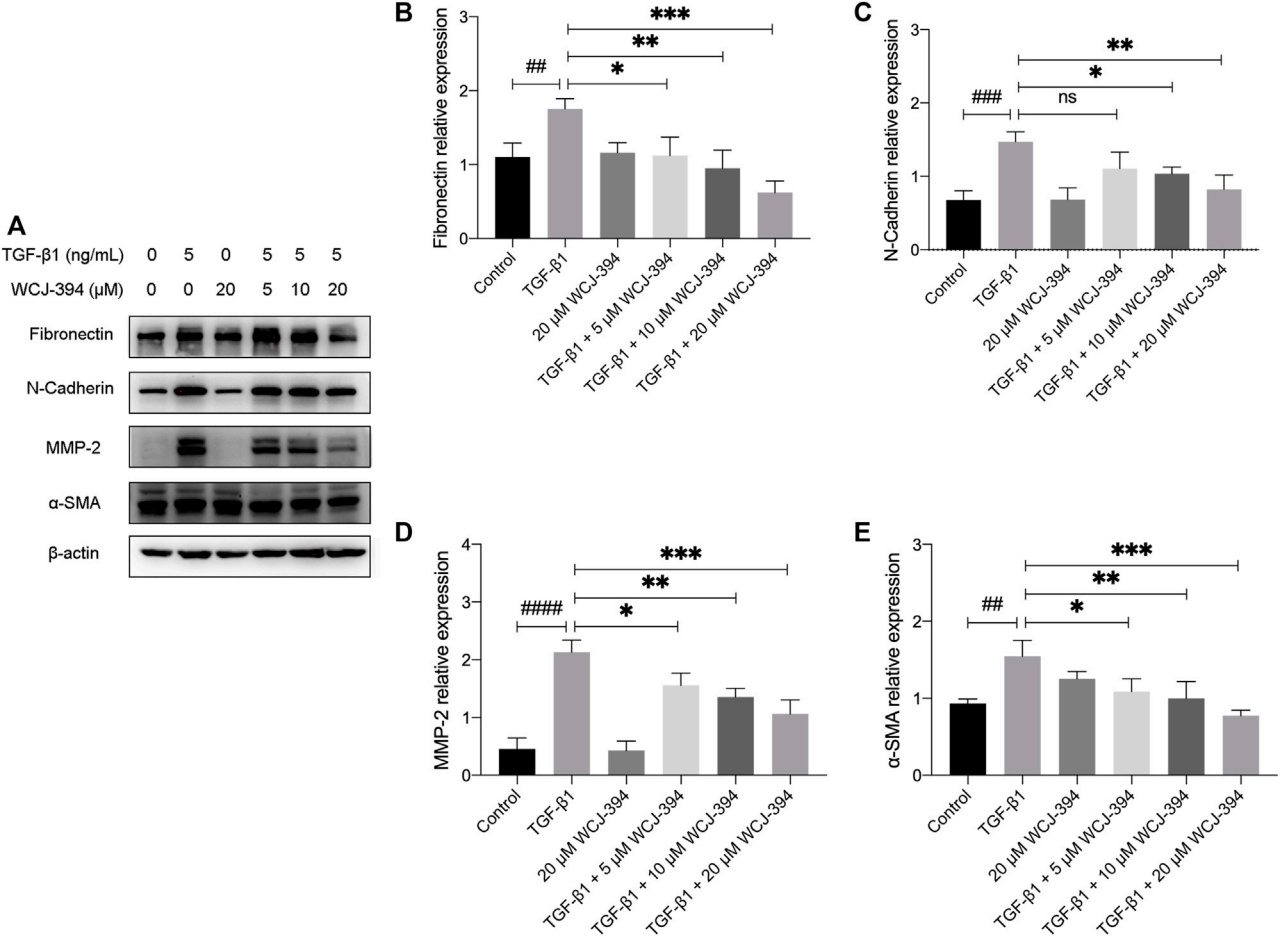
WCJ-394 repressed the expression of TGF-β1-induced mesenchymal markers in A549 cells. **(A)** The influences on fibronectin, N-cadherin, MMP-2, and α-SMA (α-smooth muscle actin) in A549 cells when treated with TGF-β1 (48 h) alone or in combination with WCJ-394 (5, 10, and 20 μΜ). **(B–E)** Expression levels of fibronectin, N-cadherin, MMP-2, and α-SMA relative to the internal control β-actin. ^##^ *p* < 0.01, ^###^ *p* < 0.001, ^####^ *p* < 0.0001 vs. the control group; ^*^ *p* < 0.05, ^**^ *p* < 0.01, ^***^ *p* < 0.001 vs. the TGF-β1 group.

As shown in Figure 5A, one of the hallmarks of EMT is the increased production of mesenchymal markers, such as fibronectin, N-cadherin, alpha-smooth muscle actin (α-SMA), and metalloproteinases (MMPs), which permits transdifferentiated cells to exhibit invasive and motile phenotypes. The effect of WCJ-394 on the expression of EMT-related proteins, including fibronectin, N-cadherin, MMP-2, and α-SMA in A549 cells induced by TGF-β1, was assessed by Western blotting. As shown in Figures 5B–E, the expression levels of those proteins were significantly (*p* < 0.01) increased in response to TGF-β1 and were decreased (*p* < 0.05) by the pretreatment of WCJ-394 in a concentration-dependent manner.

### Compound 1r (WCJ-394) Suppresses the TGF-β-Induced Cell Migration in A549 Cells

In order to investigate the effects of WCJ-394 on the TGF-β1-induced A549 cell migration, scratch wound healing and transwell assay were performed to determine the rate of wound closure and the number of migration cells, individually.

**FIGURE 6:**
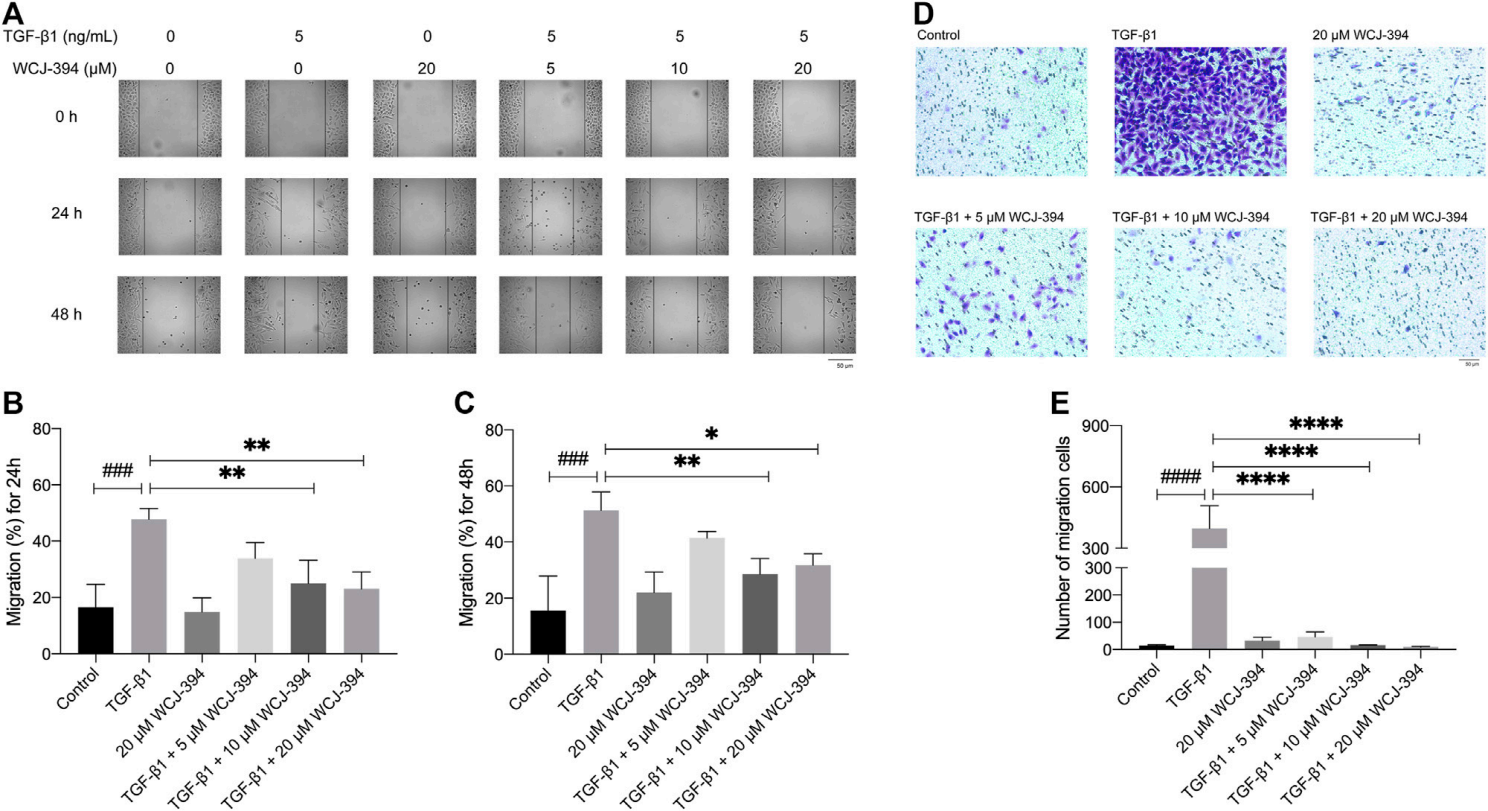
WCJ-394 inhibited the TGF-β1-induced cell migration in A549 cells. **(A)** Effects of WCJ-394 on the TGF-β1-induced A549 cell migration in a scratch wound healing-based migration assay. **(B)** The scratched wound closure of A549 cells at 24 h in the scratch wound healing assay. **(C)** The scratched wound closure of A549 cells at 48 h in the scratch wound healing assay. **(D)** Effect of WCJ-394 on the TGF-β1-induced A549 cell migration in a transwell-based migration assay. **(E)** Numbers of migration cells from different groups in the transwell assay. ^###^ *p* < 0.001, ^####^ *p* < 0.0001 vs. the control group; ^*^ *p* < 0.05, ^**^ *p* < 0.01, ^****^ *p* < 0.0001 vs. the TGF-β1 group.

In the scratch wound healing assay, the percentages of scratch widths at 24 and 48 h compared to that of 0 h were used to denote the rate of A549 cell migration. As shown in Figures 6A–C, the TGF-β1 treatment promoted the scratched wound closure. While such TGF-β1-induced cell migration was significantly inhibited by the pretreatment of WCJ-394 at 10 or 20 μΜ at 24 h, and the retardation effect of WCJ-394 on cell migration was further detected at 48 h.

Additionally, similar results were obtained in the transwell migration assay (Figures 6D,E). The number of cells, which passed through the transwell membrane, was significantly increased after TGF-β1 treatment. WCJ-394 (5, 10, and 20 μΜ) pretreatment significantly decreased the number of migrated cells in a concentration-dependent manner.

### Molecular Docking Study

**FIGURE 7:**
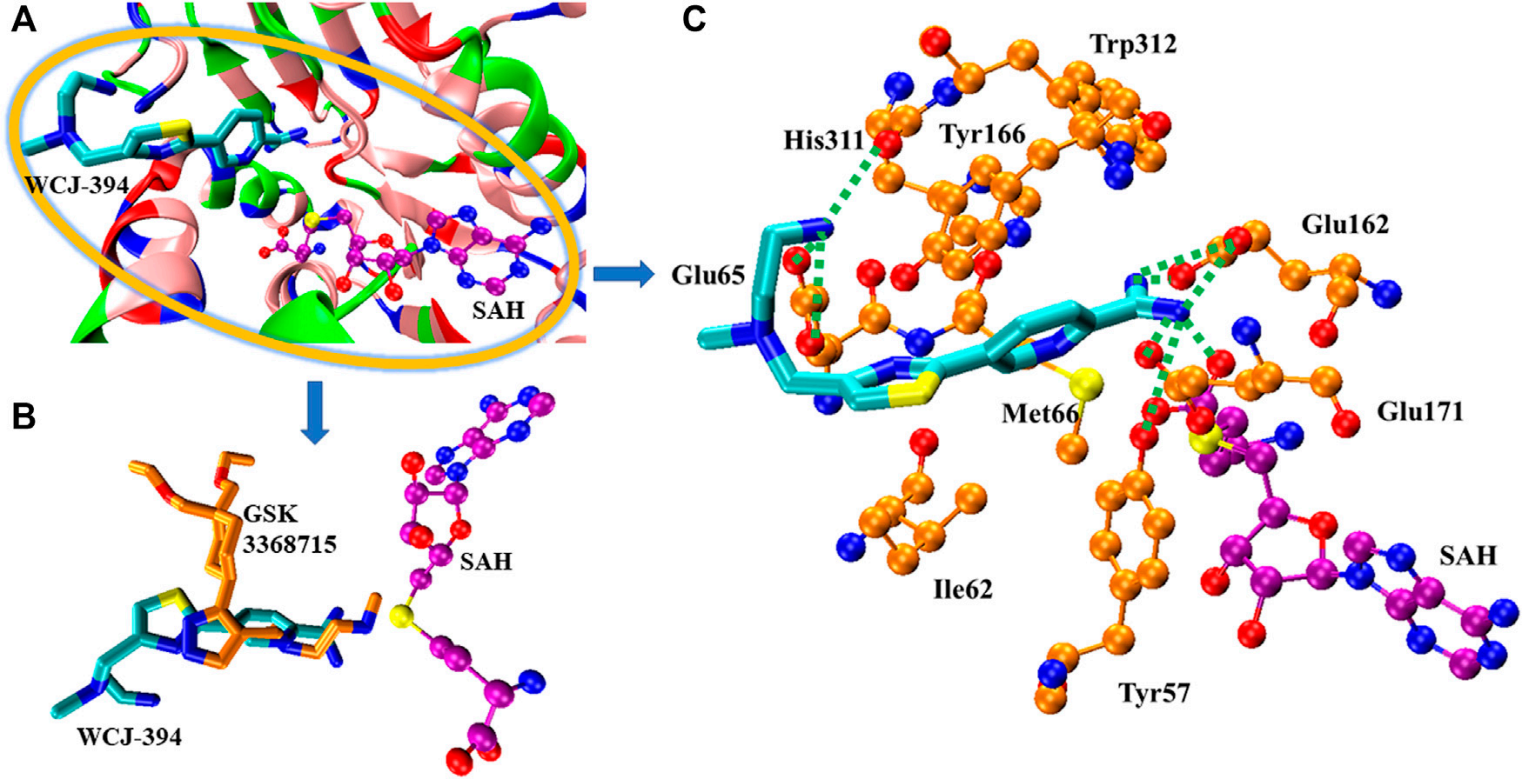
The binding of WCJ-394 with PRMT1. **(A)** The overview of the binding of WCJ-394 with PRMT1-SAH. In the current figure, the basic residues were colored in blue, the acidic residues were colored in red, the polar residues were colored in green, and the nonpolar residues were colored in pink. SAH was represented in ball–sticks model, in which the oxygen atoms were colored in red, the nitrogen atoms were colored in blue, the sulfur atoms were colored in yellow, and the carbon atoms were colored in purple unless otherwise specified. WCJ-394 was represented in sticks model, in which the carbon atoms were colored in cyan. **(B)** The comparison of the binding poses of WCJ-394 and GSK3368715 (carbon atoms in gold). **(C)** The interactions of WCJ-394 with PRMT1-SAH. The hydrogen bonds were represented in dotted lines, and the key residues were represented in ball–sticks model and carbons were in gold.

The molecular docking was performed to study the binding pose of WCJ-394 against PRMT1. The crystal structure of PRMT1 complexed with GSK3368715 (a PRMT1 inhibitor) was recently solved, and the PDB ID is 6NT2 (Fedoriw et al., 2019), which was used for molecular docking, and the binding position of GSK3368715 was used to indicate the potential binding site. As Figure 7A shows, the molecular docking clearly indicated that WCJ-394 occupied the substrate arginine binding site and did not bind to the SAH binding site. By comparing with the binding modes of WCJ-394 and GSK3368715, it can be noticed that there are some slight differences (Figure 7B). For GSK3368715, the ethylenediamine group binds close to SAH, mimicking the guanidine group of the substrate arginine. However, for WCJ-394, the amidino group (not the ethylenediamine group) occupies the binding position of the guanidine group of the substrate arginine, which may be because the amidino group shares a higher similarity with the guanidine group. The interactions between WJC-394 and PRMT1 were analyzed, as shown in Figure 7C. It can be noticed that the amidino group of WJC-394 can form six hydrogen bonds with Glu163, Glu171, and Tyr57, and also can form strong electrostatic interactions with Glu162 and Glu171. The terminal amino group on the ethylenediamine group can form electrostatic interactions with Glu65 and can form hydrogen bonds with His311 and Glu65. The π–π interactions are mainly contributed by Trp312, Tyr166, His311, and Tyr57, and Ile62 and Met66 contribute significant hydrophobic interactions. It can also be noticed that the sulfur atom of SAH is close to the amidino group of WJC-394, which may positively contribute to the stabilization of the leading compound.

## Materials and Methods

### General Information

All reagents and solvents were purchased from the supplier and used directly in the experiment. Thin-layer chromatography (TLC) was performed on silica gel (silica gel 60 pre-coated aluminum plates from Macherey-Nagel). All ^1^H and ^13^C NMR spectra were recorded on a Bruker Avance III 400 spectrometer. The solvents used for the NMR spectrum was CDCl_3_, DMSO-*d*_6_, and D_2_O, and TMS was used as the internal standard. Mass spectra were recorded on a Thermo TSQ Quantum Access Max. HRMS was recorded on an Agilent 6545 Q-TOF LC/MS. The ionization method was ESI (Elektron Spray Ionization). Melting point was measured on a WRS-1B digital melting point apparatus, uncorrected.

### Chemistry Synthesis

General procedure A: to a solution of **2** (2.66 mmol) in dichloroethane (20 ml) were added **3a–d** (2.66 mmol) and sodium triacetoxyborohydride (5.32 mmol) at 0°C under argon and then stirred at room temperature for 12 h. The reaction solution was quenched with saturated NH_4_Cl (20 ml), extracted with CH_2_Cl_2_ (30 ml × 3), and the combined organic layers were dried with Na_2_SO_4_. The residue was purified by column chromatography on silica gel using EtOAc/petroleum ether as an eluent to give **4a–d**.

General procedure B: compound **4a** (2.88 mmol) was dissolved in a mixed solvent (toluene/EtOH/H_2_O = 2:1:1, 32 ml), and **5a–g** (4.32 mmol) was added. And then, potassium carbonate (8.64 mmol) and 1,1′-di-*tert*-butylphosphinoferrocene palladium dichloride (0.058 mmol) were added to the reaction solution. The reaction mixture was heated at 80°C for 8 h. After the reaction was completed, it was quenched with saturated NH_4_Cl (20 ml) and extracted with EtOAc (20 ml × 3). The combined organic layers were dried with Na_2_SO_4_, filtered, and concentrated. The residue was purified by column chromatography on silica gel using EtOAc/petroleum ether as an eluent to give **6a–g**.

General procedure C: to a solution of compounds **6b,c** (1.36 mmol) in isopropanol (30 ml) were added hydrazine hydrate (80%, 4 ml, 68 mmol) and Pd/C (10%, 0.30 g). The reaction solution was heated at 80°C for 5 h and filtered. The filtrated was concentrated, and the residue was purified by column chromatography on silica gel using EtOAc/petroleum ether as an eluent to give **7c,d**.

General procedure D: to a solution of compounds **6d–f** (2.98 mmol) in dry ethanol (50 ml) were added hydroxylamine hydrochloride (8.94 mmol) and triethylamine (8.94 mmol). The reaction mixture was stirred at room temperature for 29 h and concentrated. To the residue was added H_2_O (30 ml) and extracted with EtOAc (10 ml × 3). The combined organic layers were dried with Na_2_SO_4_, filtered, and concentrated. The residue was purified by column chromatography on silica gel using 2% CH_3_OH in CH_2_Cl_2_ as an eluent to give **7e–g**.

General procedure E: to a solution of compounds **6d–g** (2.71 mmol) in dry THF (20 ml) was added LiHMDS (13.55 mmol) at 0°C, and the reaction mixture was stirred at room temperature for 24 h. And then, the reaction solution was cooled to 0°C and the pH was adjusted to 2**–**3 using 2 N HCl and stirred at room temperature for 2 h. The reaction solution was concentrated and the pH was adjusted to 9–10 and extracted with CH_2_Cl_2_ (10 ml × 3). The combined organic layers were dried with Na_2_SO_4_, filtered, and concentrated to give the crude product. The crude product was dissolved in CH_2_Cl_2_ (30 ml), and triethylamine (9.75 mmol) was added. And then, (Boc)_2_O (6.50 mmol) was added at 0°C and the reaction solution was stirred at room temperature for 15 h. After the reaction was completed, the reaction solution was washed with saturated NaHCO_3_ (10 ml) and H_2_O (10 ml). The organic layer was dried with Na_2_SO_4_, filtered, and concentrated. The residue was purified by column chromatography on silica gel using EtOAc/petroleum ether as an eluent to give **7h–k**.

General procedure F: compounds **7a–k** or **10a–d** or **18a–c** (2.00 mmol) were added to saturated hydrochloric acid in EtOH (20 ml); the resulting solution was stirred at room temperature for 5 h, and the reaction solution was concentrated. The residue was washed by Et_2_O, and the precipitate was filtered to give **1a–r**.

General procedure G: compounds **4a–d** (1.15 mmol) were dissolved in a mixed solvent (toluene/EtOH/H2O = 2:1:1, 30 ml), and **5h** (1.84 mmol) was added. And then, potassium carbonate (3.69 mmol) and 1,1′-di-tert-butylphosphinoferrocene palladium dichloride (0.06 mmol) were added to the reaction solution. The reaction solution was put into a microwave reactor and reacted in microwave for 0.5 h. After the reaction was completed, it was quenched with saturated NaCl (20 ml), and extracted with EtOAc (20 ml × 3). The combined organic layers were dried with Na_2_SO_4_, filtered, and concentrated. The residue was purified by column chromatography on silica gel using EtOAc/petroleum ether as an eluent to give **9a–d**.

General procedure H: to a solution of compounds **9a–d** (0.68 mmol) in methanol (10 ml) was added sodium methoxide (0.88 mmol) at 0°C, and the reaction solution was stirred for 0.5 h. And then, it was reacted at room temperature for 36 h, and ammonium chloride (0.88 mmol) was added and continued to stir for 24 h. After the reaction was completed, it was quenched with saturated NaCl (20 ml) and extracted with EtOAc (20 ml × 3). The combined organic layers were dried with Na_2_SO_4_, filtered, and concentrated. The residue was purified by silica gel column chromatography using CH_3_OH in CH_2_Cl_2_ as the eluent to give **10a–d**.

N-tert-Butyloxycarbonyl-N′-(5-bromofuran-2-yl-methyl)-N,N′-dimethylethylene Diamine **(4a)**

General procedure A. Yellow oil (750 mg, 81%); ^1^H NMR (400 MHz, DMSO-*d*_6_) δ 6.49 (d, *J* = 3.2 Hz, 1H, Ar*H*), 6.35 (d, *J* = 3.2 Hz, 1H, Ar*H*), 3.52 (s, 2H, C*H*_*2*_), 3.29–3.20 (m, 2H, C*H*_*2*_), 2.76 (s, 3H, C*H*_*3*_), 2.42 (m, 2H, C*H*_*2*_), 2.18 (s, 3H, C*H*_*3*_), 1.45 [s, 9H, C(C*H*_3_)_3_]; ES-MS 347.1 (M + H)^+^.

N-tert-Butoxycarbonyl-N-methyl-N′-(5-bromofuran-2-yl-methyl)-N′-cyclopropylethylenediamine **(4b)**

General procedure A. Yellow oil (900 mg, 78%); ^1^H NMR (400 MHz, DMSO-*d*_6_) δ 6.50 (d, *J* = 3.7 Hz, 1H, Ar*H*), 6.36 (dd, *J* = 7.2, 3.2 Hz, 1H, Ar*H*), 3.71 (s, 2H, C*H*_*2*_), 3.21 (m, 2H, C*H*_*2*_), 2.74 (s, C*H*_*3*_), 2.64 (m, 2H, C*H*_*2*_), 1.84 [m, 1H, C*H*(CH_2_)_2_], 1.37 [s, 9H, C(C*H*_3_)_3_], 0.44–0.41 [m, 2H, CH(C*H*_2_)_2_], 0.29–0.26 [m, 2H, CH(C*H*_2_)_2_]; ES-MS 373.1 (M + H)^+^.

N-tert-Butoxycarbonyl-N-methylene-N′-(5-bromofuran-2-yl-methyl)-piperazine **(4c)**

General procedure A. Yellow oil (1,100 mg, 80%); ^1^H NMR (400 MHz, DMSO-*d*_6_) δ 6.50 (d, *J* = 3.3 Hz, 1H, Ar*H*), 6.37 (d, *J* = 3.2 Hz, 1H, Ar*H*), 3.49 (s, 2H, C*H*_*2*_), 3.29 [t, *J* = 5.1 Hz, 4H, N(C*H*_*2*_)_2_], 2.31 [t, *J* = 5.1 Hz, 4H, N(C*H*_*2*_)_2_], 1.38 [s, 9H, C(C*H*_*3*_)_3_]; ES-MS 345.1 (M + H)^+^.

N-tert-Butoxycarbonyl-N′-(5-bromofuran-2-yl-methyl)-N′-methylethylenediamine **(4d)**

General procedure A. Yellow oil (850 mg, 50%); ^1^H NMR (400 MHz, DMSO-*d*_6_) δ 6.67 (t, *J* = 5.8 Hz, 1H, N*H*), 6.49 (d, *J* = 3.2 Hz, 1H, Ar*H*), 6.35 (d, *J* = 3.3 Hz, 1H, Ar*H*), 3.51 (s, 2H, C*H*_*2*_), 3.01 (m, 2H, C*H*_*2*_), 2.35 (m, 2H, C*H*_*2*_), 2.14 (s, 3H, C*H*_*3*_), 1.37 [s, 9H, C(C*H*_*3*_)_3_]; ES-MS 333.1 (M + H)^+^.

N-tert-Butyloxycarbonyl-N′-[5-(4-methoxycarbonylphenyl)-furan-2-yl-methyl]-N,N′-dimethylethylenediamine **(6a)**

General procedure B. Yellow oil (2.40 g, 82%); ^1^H NMR (400 MHz, CDCl_3_) δ 8.08–8.03 (m, 2H, Ar*H*), 7.77–7.69 (m, 2H, Ar*H*), 6.76 (d, *J* = 3.3 Hz, 1H, Ar*H*), 6.36 (d, *J* = 3.4 Hz, 1H, Ar*H*), 3.95 (s, 3H, C*H*_*3*_), 3.73 (s, 2H, C*H*_*2*_), 3.41 (m, 2H, C*H*_*2*_), 2.90 (s, 3H, C*H*_*3*_), 2.61 (m, 2H, C*H*_*2*_), 2.39 (s, 3H, C*H*_*3*_), 1.46 [s, 9H, (C*H*_*3*_)_3_]; ES-MS 403.3 (M + H)^+^.

N-tert-Butyloxycarbonyl-N′-[5-(4-nitrophenyl)-furan-2-yl-methyl]-N,N′-dimethyl Ethylenediamine **(6b)**

General procedure B. Yellow oil (0.42 g, 75%); ^1^H NMR (400 MHz, DMSO-*d*_6_) δ 8.32–8.23 (m, 2H, Ar*H*), 7.91 (d, *J* = 8.6 Hz, 2H, Ar*H*), 7.27 (d, *J* = 3.3 Hz, 1H, Ar*H*), 6.52 (d, *J* = 3.4 Hz, 1H, Ar*H*), 3.66 (s, 2H, C*H*_*2*_), 3.35 (m, 2H, C*H*_*2*_), 2.78 (s, 3H, C*H*_*3*_), 2.48 (m, 2H, C*H*_*2*_), 2.26 (s, 3H, C*H*_*3*_), 1.35 [s, 9H, (C*H*_*3*_)_3_]; ES-MS 390.2 (M + H)^+^.

N-tert-Butyloxycarbonyl-N′-[5-(3-nitrophenyl)-furan-2-yl-methyl]-N,N′-dimethyl Ethylenediamine **(6c)**

General procedure B. Yellow oil (0.66 g, 73%); ^1^H NMR (400 MHz, DMSO-*d*_6_) δ 8.41 (t, *J* = 2.0 Hz, 1H, Ar*H*), 8.11 (dd, *J* = 8.3, 2.3 Hz, 2H, Ar*H*), 7.72 (t, *J* = 8.0 Hz, 1H, Ar*H*), 7.20 (d, *J* = 3.3 Hz, 1H, Ar*H*), 6.48 (d, *J* = 3.3 Hz, 1H, Ar*H*), 3.66 (s, 2H, C*H*_*2*_), 3.28 (m, 2H, C*H*_*2*_), 2.79 (s, 3H, C*H*_*3*_), 2.48 (m, 2H, C*H*_*2*_), 2.26 (s, 3H, C*H*_*3*_), 1.35 [s, 9H, (C*H*_*3*_)_3_]; ES-MS 390.2 (M + H)^+^.

N-tert-Butyloxycarbonyl-N′-[5-(4-cyanophenyl)-furan-2-yl-methyl]-N,N′-dimethylethylenediamine **(6d)**

General procedure B. Yellow oil (1.10 g, 89%); ^1^H NMR (400 MHz, DMSO-*d*_6_) δ 7.89–7.80 (m, 4H, Ar*H*), 7.18 (d, *J* = 3.3 Hz, 1H, Ar*H*), 6.48 (d, *J* = 3.4 Hz, 1H, Ar*H*), 3.64 (s, 2H, C*H*_*2*_), 3.27 (m, 2H, C*H*_*2*_), 2.78 (s, 3H, C*H*_*3*_), 2.47 (m, 2H, C*H*_*2*_), 2.25 (s, 3H, C*H*_*3*_), 1.35 [s, 9H, (C*H*_*3*_)_3_]; ES-MS 370.2 (M + H)^+^.

N-tert-Butyloxycarbonyl-N′-[5-(3-fluoro-4-cyanophenyl)-furan-2-yl-methyl]-N,N′-dimethylethylenediamine **(6e)**

General procedure B. Yellow oil (0.68 g, 76%); ^1^H NMR (400 MHz, DMSO-*d*_6_) δ 7.95 (dd, *J* = 8.2, 6.9 Hz, 1H, Ar*H*), 7.77 (d, *J* = 10.9 Hz, 1H, Ar*H*), 7.67 (d, *J* = 8.2 Hz, 1H, Ar*H*), 7.29 (d, *J* = 3.4 Hz, 1H, Ar*H*), 6.51 (d, *J* = 3.4 Hz, 1H, Ar*H*), 3.65 (s, 2H, C*H*_*2*_), 3.33–3.23 (m, 2H, C*H*_*2*_), 2.77 (s, 3H, C*H*_*3*_), 2.47 (m, 2H, C*H*_*2*_), 2.25 (s, 3H, C*H*_*3*_), 1.35 [s, 9H, (C*H*_*3*_)_3_]; ES-MS 388.2 (M + H)^+^.

N-tert-Butyloxycarbonyl-N′-[5-(3-cyanophenyl)-furan-2-yl-methyl]-N,N′-dimethylethylenediamine **(6f)**

General procedure B. Yellow oil (0.94 g, 72%); ^1^H NMR (400 MHz, DMSO-*d*_6_) δ 8.12 (d, *J* = 1.7 Hz, 1H, Ar*H*), 7.97 (d, *J* = 7.9 Hz, 1H, Ar*H*), 7.72 (dt, *J* = 7.7, 1.4 Hz, 1H, Ar*H*), 7.62 (t, *J* = 7.8 Hz, 1H, Ar*H*), 7.11 (d, *J* = 3.3 Hz, 1H, Ar*H*), 6.45 (d, *J* = 3.3 Hz, 1H, Ar*H*), 3.63 (s, 2H, C*H*_*2*_), 3.32 (m, 2H, C*H*_*2*_), 2.77 (m, 3H, C*H*_*3*_), 2.49 (m, 2H, C*H*_*2*_), 2.25 (s, 3H, C*H*_*3*_), 1.35 [s, 9H, (C*H*_*3*_)_3_]; ES-MS 370.2 (M + H)^+^.

N-tert-Butyloxycarbonyl-N′-[5-(3-cyano-4-chlorophenyl)-furan-2-yl-methyl]-N,N′-dimethylethylenediamine **(6g)**

General procedure B. Yellow oil (0.88 g, 76%); ^1^H NMR (400 MHz, DMSO-*d*_6_) δ 8.25 (d, *J* = 2.2 Hz, 1H, Ar*H*), 7.97 (dd, *J* = 8.6, 2.2 Hz, 1H, Ar*H*), 7.78 (d, *J* = 8.6 Hz, 1H, Ar*H*), 7.15 (d, *J* = 3.3 Hz, 1H, Ar*H*), 6.46 (d, *J* = 3.4 Hz, 1H, Ar*H*), 3.63 (s, 2H, C*H*_*2*_), 3.27 (m, 2H, C*H*_*2*_), 2.77 (s, 3H, C*H*_*3*_), 2.47 (m, 2H, C*H*_*2*_), 2.25 (s, 3H, C*H*_*3*_), 1.35 [s, 9H, (C*H*_*3*_)_3_]; ES-MS 404.2 (M + H)^+^.

N-tert-Butyloxycarbonyl-N′-[5-(4-formamide-phen-1-yl)-furan-2-yl-methyl]-N,N′-dimethylethylenediamine **(7a)**

To a solution of compound **6a** (562 mg, 1.40 mmol) in DMF (2 ml) in the sealed tube were added sodium ethoxide (95 mg, 1.40 mmol) and formamide (1 ml, 24.80 mmol). The reaction solution was heated at 100°C for 2 h. After cooling, to the reaction solution was added saturated NaHCO_3_ (10 ml) and extracted with CH_2_Cl_2_ (10 ml × 3). The combined organic layers were dried with Na_2_SO_4_, filtered, and concentrated. The residue was purified by column chromatography on silica gel using 3% CH_3_OH in CH_2_Cl_2_ as an eluent to give **7a** as a yellow oil (390 mg, 72%); ^1^H NMR (400 MHz, CDCl_3_) δ 7.89–7.80 (m, 2H, Ar*H*), 7.75–7.68 (m, 2H, Ar*H*), 6.72 (d, *J* = 3.3 Hz, 1H, Ar*H*), 6.33 (d, *J* = 3.4 Hz, 1H, Ar*H*), 6.09 (s, 3H, N*H*_*2*_), 3.68 (s, 2H, C*H*_*2*_), 3.36 (m, 2H, C*H*_*2*_), 2.88 (s, 3H, C*H*_*3*_), 2.59 (m, 2H, C*H*_*2*_), 2.37 (s, 3H, C*H*_*3*_), 1.46 [s, 9H, (C*H*_*3*_)_3_]; ES-MS 388.2 (M + H)^+^.

N-tert-Butyloxycarbonyl-N′-[5-(4-formylhydrazide-phen-1-yl)-furan-2-yl-methyl]-N,N′-dimethylethylenediamine **(7b)**

To a solution of compound **6a** (510 mg, 1.27 mmol) in ethanol (30 ml) was added hydrazine hydrate (80%, 5.40 ml, 101.60 mmol). The reaction solution was heated at 80°C for 30 h. After cooling, to the reaction solution was added H_2_O (10 ml) and extracted with CH_2_Cl_2_ (10 ml × 3). The combined organic layers were dried with Na_2_SO_4_, filtered, and concentrated. The residue was purified by column chromatography on silica gel using 4% CH_3_OH in CH_2_Cl_2_ as an eluent to give **7b** as a colorless oil (310 mg, 61%); ^1^H NMR (400 MHz, DMSO-*d*_6_) δ 9.80 (s, 1H, N*H*), 7.87 (d, *J* = 8.5 Hz, 2H, Ar*H*), 7.72 (d, *J* = 8.2 Hz, 2H, Ar*H*), 7.02 (d, *J* = 3.3 Hz, 1H, Ar*H*), 6.43 (d, *J* = 3.3 Hz, 1H, Ar*H*), 4.50 (s, 2H, N*H*_*2*_), 3.63 (s, 2H, C*H*_*2*_), 3.30–3.26 (m, 2H, C*H*_*2*_), 2.78 (s, 3H, C*H*_*3*_), 2.48 (m, 2H, C*H*_*2*_), 2.25 (s, 3H, C*H*_*3*_), 1.36 [s, 9H, (C*H*_*3*_)_3_]; ES-MS 403.2 (M + H)^+^.

N-tert-Butyloxycarbonyl-N′-[5-(4-aminophenyl)-furan-2-yl-methyl]-N,N′-dimeth-ylethylenediamine **(7c)**

General procedure C. Yellow oil (0.33 g, 67%); ^1^H NMR (400 MHz, DMSO-*d*_6_) δ 7.32 (d, *J* = 8.4 Hz, 2H, Ar*H*), 6.63–6.53 (m, 2H, Ar*H*), 6.46 (d, *J* = 3.2 Hz, 1H, Ar*H*), 6.27 (d, *J* = 3.2 Hz, 1H, Ar*H*), 5.28 (s, 2H, N*H*_*2*_), 3.54 (s, 2H, C*H*_*2*_) 3.31–3.23 (m, 2H, C*H*_*2*_), 2.77 (s, 3H, C*H*_*3*_), 2.45 (m, 2H, C*H*_*2*_), 2.22 (s, 3H, C*H*_*3*_), 1.37 [s, 9H, (C*H*_*3*_)_3_]; ES-MS 360.2 (M + H)^+^.

N-tert-Butyloxycarbonyl-N′-[5-(3-aminophenyl)-furan-2-yl-methyl]-N,N′-dimeth-ylethylenediamine **(7d)**

General procedure C. Yellow oil (0.45 g, 79%); ^1^H NMR (400 MHz, DMSO-*d*_6_) δ 7.03 (t, *J* = 7.8 Hz, 1H, Ar*H*), 6.90–6.79 (m, 2H, Ar*H*), 6.67 (d, *J* = 3.2 Hz, 1H, Ar*H*), 6.47 (ddd, *J* = 8.0, 2.3, 1.0 Hz, 1H, Ar*H*), 6.34 (d, *J* = 3.3 Hz, 1H, Ar*H*), 5.17 (s, 2H, N*H*_*2*_), 3.59 (s, 2H, C*H*_*2*_), 3.33–3.22 (m, 2H, C*H*_*2*_), 2.78 (s, 3H, C*H*_*3*_), 2.47 (m, 2H, C*H*_*2*_), 2.24 (s, 3H, C*H*_*3*_), 1.37 [s, 9H, (C*H*_*3*_)_3_]; ES-MS 360.2 (M + H)^+^.

N-tert-Butyloxycarbonyl-N′-[5-(4-N-hydroxyamidino-phen-1-yl)-furan-2-yl-methyl]-N,N′-dimethylethylenediamine **(7e)**

General procedure D. Yellow oil (0.65 g, 54%); ^1^H NMR (400 MHz, DMSO-*d*_6_) δ 9.69 (s, 1H, O*H*), 7.71 (d, *J* = 8.6 Hz, 2H, Ar*H*), 7.65 (d, *J* = 8.5 Hz, 2H, Ar*H*), 6.92 (d, *J* = 3.3 Hz, 1H, Ar*H*), 6.40 (d, *J* = 3.3 Hz, 1H, Ar*H*), 5.83 (s, 2H, N*H*_*2*_), 3.62 (s, 2H, C*H*_*2*_), 3.28 (m, 2H, C*H*_*2*_), 2.78 (s, 3H, C*H*_*3*_), 2.48 (m, 2H, C*H*_*2*_), 2.25 (s, 3H, C*H*_*3*_), 1.36 [s, 9H, (C*H*_*3*_)_3_]; ES-MS 403.2 (M + H)^+^.

N-tert-Butyloxycarbonyl-N′-[5-(3-fluoro-4-N-hydroxyamidino-phen-1-yl)-furan-2-yl-methyl]-N,N′-dimethylethylenediamine **(7f)**

General procedure D. Yellow oil (0.50 g, 54%); ^1^H NMR (400 MHz, DMSO-*d*_6_) δ 9.71 (s, 1H, O*H*), 7.59–7.45 (m, 3H, Ar*H*), 7.05 (d, *J* = 3.3 Hz, 1H, Ar*H*), 6.43 (d, *J* = 3.3 Hz, 1H, Ar*H*), 5.83 (s, 2H, N*H*_*2*_), 3.63 (s, 2H, C*H*_*2*_), 3.28 (m, 2H, C*H*_*2*_), 2.77 (s, 3H, C*H*_*3*_), 2.47 (m, 2H, C*H*_*2*_), 2.25 (s, 3H, C*H*_*3*_), 1.36 [s, 9H, (C*H*_*3*_)_3_]; ES-MS 421.2 (M + H)^+^.

N-tert-Butyloxycarbonyl-N′'-[5-(3-N-hydroxyamidino-phen-1-yl)-furan-2-yl-methyl]-N,N′-dimethylethylenediamine **(7g)**

General procedure D. Yellow oil (0.44 g, 63%); ^1^H NMR (400 MHz, DMSO-*d*_6_) δ 9.70 (s, 1H, O*H*), 7.97 (t, *J* = 1.7 Hz, 1H, Ar*H*), 7.66 (d, *J* = 7.8 Hz, 1H, Ar*H*), 7.57 (dt, *J* = 7.9, 1.3 Hz, 1H, Ar*H*), 7.41 (t, *J* = 7.8 Hz, 1H, Ar*H*), 6.91 (d, *J* = 3.2 Hz, 1H, Ar*H*), 6.41 (d, *J* = 3.3 Hz, 1H, Ar*H*), 5.90 (s, 2H, N*H*_*2*_), 3.62 (s, 2H, C*H*_*2*_), 3.28 (m, 2H, C*H*_*2*_), 2.78 (s, 3H, C*H*_*3*_), 2.47 (m, 2H, C*H*_*2*_), 2.25 (s, 3H, *CH*_*3*_), 1.36 [s, 9H, (C*H*_*3*_)_*3*_]; ES-MS 403.2 (M + H)^+^.

N-tert-Butyloxycarbonyl-N′-[5-(4-N-tert-butyloxycarbonylamidino-phen-1-yl)-furan-2-yl-methyl]-N,N′-dimethylethylenediamine **(7h)**

General procedure E. Yellow oil (0.24 g, 92%); ^1^H NMR (400 MHz, DMSO-*d*_6_) δ 9.07 (s, 2H, N*H*), 8.06–7.98 (m, 2H, Ar*H*), 7.75 (d, *J* = 8.6 Hz, 2H, Ar*H*), 7.06 (d, *J* = 3.3 Hz, 1H, Ar*H*), 6.45 (d, *J* = 3.3 Hz, 1H, Ar*H*), 3.64 (s, 2H, C*H*_*2*_), 3.28 (m, 2H, C*H*_*2*_), 2.78 (s, 3H, C*H*_*3*_), 2.48 (m, 2H, C*H*_*2*_), 2.26 (s, 3H, C*H*_*3*_), 1.46 [s, 9H, C(C*H*_*3*_)_3_], 1.36 (s, 9H, C(C*H*_*3*_)_3_); ES-MS 487.3 (M + H)^+^.

N-tert-Butyloxycarbonyl-N′-[5-(3-fluoro-4-N-tert-butyloxycarbonylamidino-phen-1-yl)-furan-2-yl-methyl]-N,N′-dimethylethylenediamine **(7i)**

General procedure E. Yellow oil (0.30 g, 79%); ^1^H NMR (400 MHz, DMSO-*d*_6_) δ 8.94 (s, 1H, N*H*), 8.57 (s, 1H, N*H*), 7.67 (t, *J* = 8.0 Hz, 1H, Ar*H*), 7.60–7.52 (m, 2H, Ar*H*), 7.13 (d, *J* = 3.3 Hz, 1H, Ar*H*), 6.46 (d, *J* = 3.4 Hz, 1H, Ar*H*), 3.64 (s, 2H, C*H*_*2*_), 3.28 (m, 2H, C*H*_*2*_), 2.78 (s, 3H, C*H*_*3*_), 2.47 (m, 2H, C*H*_*2*_), 2.25 (s, 3H, C*H*_*3*_), 1.43 [s, 9H, C(C*H*_*3*_)_3_], 1.39 [s, 9H, C(C*H*_*3*_)_3_]; ES-MS 505.3 (M + H)^+^.

N-tert-Butyloxycarbonyl-N′-[5-(3-N-tert-butyloxycarbonylamidino-phen-1-yl)-furan-2-yl-methyl]-N,N′-dimethylethylenediamine **(7j)**

General procedure E. Yellow oil (0.28 g, 84%); ^1^H NMR (400 MHz, DMSO-*d*_6_) δ 9.07 (s, 2H, N*H*), 8.20 (s, 1H, Ar*H*), 7.89–7.79 (m, 2H, Ar*H*), 7.51 (t, *J* = 7.8 Hz, 1H, Ar*H*), 6.97 (d, *J* = 3.3 Hz, 1H, Ar*H*), 6.43 (d, *J* = 3.3 Hz, 1H, Ar*H*), 3.64 (s, 2H, C*H*_*2*_), 3.29 (m, 2H, C*H*_*2*_), 2.78 (s, 3H, C*H*_*3*_), 2.48 (m, 2H, C*H*_*2*_), 2.26 (s, 3H, C*H*_*3*_), 1.46 [s, 9H, C(C*H*_*3*_)_3_], 1.36 [s, 9H, C(C*H*_*3*_)_3_]; ES-MS 487.3 (M + H)^+^.

N-tert-Butyloxycarbonyl-N′-[5-(3-N-tert-butyloxycarbonylamidino-4-chloro-phen-1-yl)-furan-2-yl-methyl]-N,N′-dimethylethylenediamine **(7k)**

General procedure E. Yellow oil (0.22 g, 32%); ^1^H NMR (400 MHz, DMSO-*d*_6_) δ 8.67 (s, 1H, N*H*), 8.50 (s, 1H, N*H*), 7.73–7.68 (m, 1H, Ar*H*), 7.65 (p, *J* = 3.8 Hz, 1H, Ar*H*), 7.53 (d, *J* = 8.4 Hz, 1H, Ar*H*), 7.05 (d, *J* = 3.3 Hz, 1H, Ar*H*), 6.42 (d, *J* = 3.4 Hz, 1H, Ar*H*), 3.62 (s, 2H, C*H*_*2*_), 3.27 (m, 2H, C*H*_*2*_), 2.77 (s, 3H, C*H*_*3*_), 2.47 (m, 2H, C*H*_*2*_), 2.24 (s, 3H, C*H*_*3*_), 1.44 [s, 9H, C(C*H*_*3*_)_3_], 1.27 [s, 9H, C(C*H*_*3*_)_3_]; ES-MS 521.3 (M + H)^+^.

N-tert-Butoxycarbonyl-N′-[5-(2-cyano-5-pyridyl)furan-2-yl-methyl]-N,N′-dimethylethylenediamine **(9a)**

General procedure G. Yellow oil (0.50 g, 59%); ^1^H NMR (400 MHz, DMSO-*d*_6_) δ 9.07 (d, *J* = 2.1 Hz, 1H, Ar*H*), 8.21 (dd, *J* = 8.2, 2.1 Hz, 1H, Ar*H*), 8.06 (d, *J* = 8.2 Hz, 1H, Ar*H*), 7.34 (d, *J* = 3.4 Hz, 1H, Ar*H*), 6.54 (d, *J* = 3.4 Hz, 1H, Ar*H*), 3.67 (s, 2H, C*H*_2_), 3.28 (m, 2H, C*H*_2_), 2.78 (s, 3H, C*H*_3_), 2.48 (m, 2H, C*H*_2_), 2.26 (s, 3H, C*H*_3_), 1.35 [s, 9H, (C*H*_*3*_)_*3*_]; ES-MS 371.2 (M + H)^+^.

N-tert-Butoxycarbonyl-N-methyl-N′-[5-(2-cyano-5-pyridyl)furan-2-yl-methyl]-N′-cyclopropylethylenediamine **(9b)**

General procedure G. Yellow oil (0.50 g, 59%); ^1^H NMR (400 MHz, DMSO-*d*_6_) δ 9.07 (d, *J* = 2.2 Hz, 1H, Ar*H*), 8.21 (d, *J* = 8.2 Hz, 1H, Ar*H*), 8.06 (d, *J* = 8.3 Hz, 1H, Ar*H*), 7.35 (d, *J* = 3.4 Hz, 1H, Ar*H*), 6.56 (d, *J* = 3.4 Hz, 1H, Ar*H*), 3.84 (s, 2H, C*H*_2_), 3.28 (m, 2H, C*H*_2_), 2.76 (s, 3H, C*H*_3_), 2.69 (m, 2H, C*H*_2_), 1.93 [m, 1H, C*H*(CH_2_)_2_], 1.37 [s, 9H, (C*H*_3_)_3_], 0.47–0.44 [m, 2H, CH(C*H*_2_)_2_], 0.34–0.28 [m, 2H, CH(C*H*_2_)_2_]; ES-MS 397.2 (M + H)^+^.

N-tert-Butoxycarbonyl-N-methylene-N′-[5-(2-cyano-5-pyridyl)furan-2-yl-methyl]-piperazine **(9c)**

General procedure G. Yellow oil (0.65 g, 55%); ^1^H NMR (400 MHz, DMSO-*d*_6_) δ 9.08 (d, *J* = 2.2 Hz, 1H, Ar*H*), 8.23 (dd, *J* = 8.3, 2.2 Hz, 1H, Ar*H*), 8.07 (d, *J* = 8.2 Hz, 1H, Ar*H*), 7.35 (d, *J* = 3.4 Hz, 1H, Ar*H*), 6.56 (d, *J* = 3.4 Hz, 1H, Ar*H*), 3.63 (s, 2H, C*H*_2_), 2.67 [t, *J* = 4.8 Hz, 4H, N (C*H*_2_)_2_], 2.39 [t, *J* = 5.0 Hz, 4H, N(C*H*_2_)_2_], 1.38 [s, 9H, (C*H*_3_)_3_]; ES-MS 369.2 (M + H)^+^.

N-tert-Butoxycarbonyl-N′-[5-(2-cyano-5-pyridyl)furan-2-yl-methyl]-N′-methylethylenediamine **(9d)**

General procedure G. Yellow oil (0.40 g, 45%);^1^H NMR (400 MHz, DMSO-*d*_6_) δ 9.08 (d, *J* = 2.2 Hz, 1H, Ar*H*), 8.23 (dd, *J* = 8.2, 2.3 Hz, 1H, Ar*H*), 8.06 (d, *J* = 8.2 Hz, 1H, Ar*H*), 7.34 (d, *J* = 3.4 Hz, 1H, Ar*H*), 6.70 (t, *J* = 5.9 Hz, 1H, N*H*), 6.56 (d, *J* = 3.4 Hz, 1H, Ar*H*), 3.70 (s, 2H, C*H*_2_), 3.08 (m, 2H, C*H*_2_), 2.47 (m, 2H, C*H*_2_), 2.26 (s, 3H, C*H*_3_), 1.36 [s, 9H, (C*H*_3_)_3_]; ES-MS 357.2 (M + H)^+^.

N-tert-Butoxycarbonyl-N′-[5-(2-amidino-5-pyridyl)furan-2-yl-methyl]-N,N′-dimethylethylenediamine **(10a)**

General procedure H. Yellow oil (0.12 g, 80%); ^1^H NMR (400 MHz, DMSO-*d*_6_) 9.55 (s, 1H, N*H*), 9.32 (s, 2H, N*H*_2_), 9.10 (s, 1H, Ar*H*), 8.35 (d, *J* = 1.6 Hz, 2H, Ar*H*), 7.39 (d, *J* = 3.4 Hz, 1*H*, Ar*H*), 6.56 (d, *J* = 3.4 Hz, 1H, Ar*H*), 3.67 (s, 2H, C*H*_2_), 3.28 (m, 2H, C*H*_2_), 2.78 (s, 3H, C*H*_3_), 2.48 (m, 2H, C*H*_2_), 2.27 (s, 3H, C*H*_3_), 1.35 [s, 9H, (C*H*_3_)_3_]; ES-MS 388.2 (M + H)^+^.

N-tert-Butoxycarbonyl-N-methyl-N′-[5-(2-amidino-5-pyridyl)furan-2-yl-methyl]-N′-cyclopropylethylenediamine **(10b)**

General procedure H. Yellow oil (0.12 g, 55%); ^1^H NMR (400 MHz, DMSO-*d*_6_) δ 9.62 (s, 1H, N*H*), 9.22 (s, 2H, N*H*_2_), 9.09 (d, *J* = 2.1 Hz, 1H, Ar*H*), 8.48 (d, *J* = 8.4 Hz, 1H, Ar*H*), 8.31 (dd, *J* = 8.4, 2.2 Hz, 1H, Ar*H*), 7.39 (d, *J* = 3.4 Hz, 1H, Ar*H*), 6.55 (d, *J* = 3.4 Hz, 1H, Ar*H*), 3.84 (s, 2H, C*H*_2_), 3.31–3.22 (m, 2H, C*H*_2_), 2.75 (s, 3H, C*H*_3_), 2.69 (m, 2H, C*H*_2_), 1.99–1.86 [m, 1H, C*H*(CH_2_)_2_], 1.35 [s, 9H, (C*H*_3_)_3_], 0.46 [m, 2H, CH(C*H*_2_)_2_], 0.30 [m, 2H, CH(C*H*_2_)_2_]; ES-MS 414.3(M + H)^+^.

N-tert-Butoxycarbonyl-N-methylene-N′-[5-(2-amidino-5-pyridyl)furan-2-yl-methyl]-piperazine **(10c)**

General procedure H. Yellow powder (0.18 g, 52%); ^1^H NMR (400 MHz, DMSO-*d*_6_) δ 9.51 (s, 2H, N*H*_2_), 9.17 (s, 1H, N*H*), 9.12 (dd, *J* = 2.2, 0.8 Hz, 1H, Ar*H*), 8.36 (dd, *J* = 8.4, 2.2 Hz, 1H, Ar*H*), 8.31 (d, *J* = 8.4 Hz, 1H, Ar*H*), 7.39 (d, *J* = 3.4 Hz, 1H, Ar*H*), 6.58 (d, *J* = 3.4 Hz, 1H, Ar*H*), 3.64 (s, 2H, C*H*_2_), 3.32 [s, 4H, N(C*H*_2_)_2_], 2.41 [s, 4H, N(C*H*_2_)_2_], 1.38 [s, 9H, (C*H*_3_)_3_]; ES-MS 386.2 (M + H)^+^.

N-tert-Butoxycarbonyl-N′-[5-(2-amidino-5-pyridyl)furan-2-yl-methyl]-N′-methylethylenediamine **(10d)**

General procedure H. Yellow powder (0.18 g, 52%); ^1^H NMR (400 MHz, DMSO-*d*_6_) δ 9.59 (s, 2H, N*H*_2_), 9.43 (s, 1H, N*H*), 9.11 (d, *J* = 2.1 Hz, 1H, Ar*H*), 8.42-8.31 (m, 2H, Ar*H and* Ar*H*), 7.40 (d, *J* = 3.4 Hz, 1H, Ar*H*), 6.74 (d, *J* = 6.1 Hz, 1H, N*H*), 6.56 (d, *J* = 3.4 Hz, 1H, Ar*H*), 3.67 (s, 2H, C*H*_2_), 3.07 (m, 2H, C*H*_2_), 2.43 (m, 2H, C*H*_2_), 2.23 (s, 3H, C*H*_3_), 1.35 [s, 9H, (C*H*_3_)_3_]; ES-MS 374.2 (M + H)^+^.

Ethyl 2-Aminothiazole-4-carboxylate **(13)**

To a solution of thiourea **12** (5.00 g, 65.69 mmol) in absolute ethanol (70 ml) was added ethyl bromopyruvate **11** (12.80 g, 72.26 mmol), and the reaction mixture was stirred at 78°C for 2.5 h. The reaction mixture was cooled to 0°C, crystallized, and filtered to give product **13** as a yellow solid (10.52 g, 93%). Without purification, it was proceeded directly to the next reaction.

Ethyl 2-Bromothiazole-4-carboxylate **(14)**

To a solution of **13** (5.00 g, 29.03 mmol) and CuBr_2_ (9.63 g, 43.11 mmol) in acetonitrile (150 ml) was added tert-butyl nitrite (5.70 ml) slowly to the reaction solution at 0°C, and then the reaction solution was reacted for 2.5 h. After the reaction was completed, CH_2_Cl_2_/H_2_O (50 ml/50 ml) was added, and then concentrated hydrochloric acid was added dropwise to adjust the pH to 1. The solution was extracted with CH_2_Cl_2_ (30 ml × 3), and the combined organic layers were dried with Na_2_SO_4_, filtered, and concentrated. The residue was purified by silica gel column chromatography using EtOAc/petroleum ether as an eluent to give **14** as a white solid (2.05 g, 30%), m.p. 66.4–68.2°C; ^1^H NMR (400 MHz, DMSO-*d*_6_) δ 8.54 (s, 1H, Ar*H*), 4.31 (q, *J* = 7.1 Hz, 2H, OC*H*_2_), 1.31 (t, *J* = 7.1 Hz, 3H, CH_2_C*H*_3_); ES-MS 236.0 (M + H)^+^.

2-Bromothiazole-4-carbaldehyde **(15)**

Compound **14** (1.08 g, 4.57 mmol) was dissolved in ethanol (35 ml), NaBH_4_ (0.35 g, 9.14 mmol) was added to the reaction solution at 0°C, and reacted for 0.5 h. The reaction solution was stirred at room temperature for 3.5 h and next at 70°C for 6 h. After the reaction was completed, it was quenched with saturated NaCl (20 ml) and extracted with EtOAc (30 ml × 3). The combined organic layers were dried with Na_2_SO_4_, filtered, and concentrated to a crude product, which was proceeded directly to the next reaction without purification.

The crude product was dissolved in anhydrous tetrahydrofuran (20 ml), and the Dess–Martin oxidant (5.47 mmol) was added; then, the reaction solution was stirred at room temperature for 2 h. The reaction solution was quenched with saturated NaCl (20 ml) and extracted with EtOAc (30 ml × 3). The organic layers were dried with Na_2_SO_4_, filtered, and concentrated. The residue was purified by column chromatography on silica gel using EtOAc/petroleum ether as an eluent to give **15** as a white solid (0.63 g, 72%), m.p. 120.4–123.6°C; ^1^H NMR (400 MHz, DMSO-*d*_6_) δ 9.83 (s, 1H, C*H*O), 8.74 (s, 1H, Ar*H*); ES-MS 191.9 (M + H)^+^.

N-tert-Butoxycarbonyl-N′-(2-bromothiazol-4-yl-methyl)-N,N′-dimethylethylenediamine **(16a)**

General procedure A. Yellow solid (1.31 g, 55%); ^1^H NMR (400 MHz, DMSO-*d*_6_) δ 7.48 (s, 1H, Ar*H*), 3.63 (s, 2H, C*H*_2_), 3.25 (m, 2H, C*H*_2_), 2.79 (s, 3H, C*H*_3_), 2.47 (m, 2H, C*H*_2_), 2.22 (s, 3H, C*H*_3_), 1.37 [s, 9H, (C*H*_3_)_3_]; ES-MS 364.1 (M + H)^+^.

N-tert-Butoxycarbonyl-N′-(2-bromothiazol-4-yl-methyl)-N′-methylethylenediamine **(16b)**

General procedure A. Yellow oil (0.73 g, 77%); ^1^H NMR (400 MHz, DMSO-*d*_6_) δ 7.51 (s, 1H, Ar*H*), 6.68 (t, *J* = 5.8 Hz, 1H, N*H*), 3.61 (s, 2H, C*H*_*2*_), 3.04 (m, 2H, C*H*_*2*_), 2.40 (m, 2H, C*H*_*2*_), 2.19 (s, 3H, C*H*_*3*_), 1.37 [s, 9H, (C*H*_*3*_)_3_]; ES-MS 350.1 (M + H)^+^.

N-tert-Butoxycarbonyl-N′-[2-(4-cyanophenyl)thiazol-4-yl-methyl]-N,N′-dimethylethylenediamine **(17a)**

General procedure B. Yellow oil (0.87 g, 87%); ^1^H NMR (400 MHz, DMSO-*d*_6_) δ 8.11 (d, *J* = 2.0 Hz, 2H, Ar*H*), 7.96 (d, *J* = 1.6 Hz, 2H, Ar*H*), 7.63 (s, 1H, Ar*H*), 3.76 (s, 2H, C*H*_2_), 3.30 (s, 3H, C*H*_3_), 2.79 (s, 3H, C*H*_3_), 2.55 (m, 2H, C*H*_2_), 2.30 (m, 2H, C*H*_2_), 1.35 [s, 9H, (C*H*_3_)_3_]; ES-MS 387.2 (M + H)^+^.

N-tert-Butoxycarbonyl-N′-[2-(2-cyano-5-pyridyl)thiazol-4-yl-methyl]-N,N′-dimethylethylenediamine **(17b)**

General procedure G. Yellow oil (0.55 g, 70%); ^1^H NMR (400 MHz, DMSO-*d*_6_) δ 9.28 (d, *J* = 2.2 Hz, 1H, Ar*H*), 8.52 (dd, *J* = 8.2, 2.3 Hz, 1H, Ar*H*), 8.16 (d, *J* = 8.2 Hz, 1H, Ar*H*), 7.72 (s, 1H, Ar*H*), 3.75 (s, 2H, C*H*_2_), 3.30 (m, 2H, C*H*_2_), 2.79 (s, 3H, C*H*_3_), 2.55 (m, 2H, C*H*_2_), 2.27 (s, 3H, C*H*_3_), 1.36 [s, 9H,(C*H*_3_)_3_]; ES-MS 388.2 (M + H)^+^.

N-tert-Butoxycarbonyl-N′-[2-(2-cyano-5-pyridyl)thiazol-4-yl-methyl]-N′-methylethylenediamine **(17c)**

General procedure G. Yellow oil (0.45 g, 65%); ^1^H NMR (400 MHz, DMSO-*d*_6_) δ 9.36 (d, *J* = 2.2 Hz, 1H, Ar*H*), 8.60 (dd, *J* = 8.2, 2.0 Hz, 1H, Ar*H*), 8.23 (d, *J* = 8.2 Hz, 1H, Ar*H*), 7.84 (s, 1H, Ar*H*), 6.75 (t, *J* = 6.0 Hz, 1H, N*H*), 3.83 (s, 2H, C*H*_2_), 3.17 (m, 2H, C*H*_2_), 2.54 (m, 2H,C*H*_2_), 2.34 (s, 3H, C*H*_3_), 1.44 [s, 9H, (C*H*_3_)_3_]; ES-MS 3374.2 (M + H)^+^.

N-tert-Butyloxycarbonyl-N′-[2-(4-N-tert-butyloxycarbonylamidino-phen-1-yl]-thiazol-4-yl-methyl)-N,N′-dimethylethylenediamine **(18a)**

General procedure E. Yellow oil (80 mg, 29%); 1H NMR (400 MHz, DMSO-*d*_6_) δ 9.07 (s, 1H, N*H*), 8.07 (d, *J* = 8.7 Hz, 2H, Ar*H*), 8.01 (d, *J* = 8.6 Hz, 2H, Ar*H*), 5.76 (s, 1H, Ar*H*), 3.73 (s, 2H, C*H*_2_), 3.29 (m, 2H, C*H*_2_), 2.79 (s, 3H, C*H*_3_), 2.29 (s, 3H, C*H*_3_), 1.45 [s, 9H, (C*H*_3_)_3_], 1.35 [s, 9H, (C*H*_3_)_3_]; ES-MS 504.2 (M + H)^+^.

N-tert-Butoxycarbonyl-N′-[2-(2-amidino-5-pyridyl)thiazol-4-yl-methyl]-N,N′-dimethylethylenediamine **(18b)**

General procedure H. Yellow oil (0.10 g, 72%); ^1^H NMR (400 MHz, DMSO-*d*_6_) δ 9.69 (s, 3H, N*H* and N*H*_2_), 9.30 (d, *J* = 2.2 Hz, 1H, Ar*H*), 8.64 (dd, *J* = 8.3, 2.3 Hz, 1H, Ar*H*), 8.52 (d, *J* = 8.4 Hz, 1H, Ar*H*), 7.78 (s, 1H, Ar*H*), 3.85 (s, 2H, C*H*_2_), 3.51 (m, 2H, C*H*_2_), 2.79 (s, 3H, C*H*_3_), 2.59 (m, 2H, C*H*_2_), 2.35 (s, 3H, C*H*_3_), 1.35 [s, 9H, (C*H*_3_)_3_]; ES-MS 405.2 (M + H)^+^.

N-tert-Butoxycarbonyl-N′-[2-(2-amidino-5-pyridyl)thiazol-4-yl-methyl]-N′-methylethylenediamine **(18c)**

General procedure H. Yellow oil (0.12 g, 70%); ^1^H NMR (400 MHz, DMSO-*d*_6_) δ 9.74 (s, 2H, N*H*_2_), 9.31 (d, *J* = 2.2 Hz, 1H, N*H*), 8.64 (dd, *J* = 8.3, 2.2 Hz, 1H, Ar*H*), 8.52 (d, *J* = 8.3 Hz, 1H, Ar*H*), 8.19 (d, *J* = 1.9 Hz, 1H, Ar*H*), 7.80 (s, 1H, Ar*H*), 6.75 (t, *J* = 5.7 Hz, 1H, N*H*), 3.82 (s, 2H, C*H*_2_), 3.11 (m, 2H, C*H*_2_), 2.59 (m, 2H,C*H*_2_), 2.30 (s, 3H, C*H*_3_), 1.35 [s, 9H, (C*H*_3_)_3_].

N′-[5-(4-Formamide-phen-1-yl)-furan-2-yl-methyl]-N,N′-dimethylethylenediamine Hydrochloride **(1a)**

General procedure F. Yellow solid (0.26 g, 52%); m.p. 212.2–214.6°C; ^1^H NMR (400 MHz, D_2_O) δ 7.66–7.58 (m, 4H, Ar*H*), 6.81 (d, *J* = 3.5 Hz, 1H, Ar*H*), 6.75 (d, *J* = 3.5 Hz, 1H, Ar*H*), 4.45 (s, 2H, C*H*_*2*_), 3.49 (m, 2H, C*H*_2_), 3.43 (m, 2H, C*H*_2_), 2.85 (s, 3H, C*H*_*3*_), 2.67 (s, 3H, C*H*_*3*_); ^13^C NMR (101 MHz, D_2_O) δ 172.27, 155.06, 142.57, 132.93, 131.86, 128.12, 124.01, 117.71, 108.55, 52.38, 49.92, 42.79, 39.91, 33.23; HRMS (ESI) m/z [M + H]^+^ calcd for C_16_H_22_N_3_O_2_^+^: 288.1707, found: 288.1706.

N′-[5-(4-Formylhydrazide-phen-1-yl)-furan-2-yl-methyl]-N,N′-dimethylethylenediamine Hydrochloride **(1b)**

General procedure F. Yellow solid (0.22 g, 84%); m.p. 246.2–246.9°C; ^1^H NMR (400 MHz, D_2_O) δ 7.82–7.73 (m, 4H, Ar*H*), 6.93 (d, *J* = 3.5 Hz, 1H, Ar*H*), 6.79 (d, *J* = 3.5 Hz, 1H, Ar*H*), 4.48 (s, 2H, C*H*_*2*_), 3.53–3.48 (m, 2H, C*H*_*2*_), 3.45–3.41 (m, 2H, C*H*_*2*_), 2.86 (s, 3H, C*H*_*3*_), 2.67 (s, 3H, C*H*_*3*_); ^13^C NMR (101 MHz, D_2_O) δ 168.07, 154.70, 142.75, 133.75, 128.76, 128.24, 124.12, 117.70, 108.96, 52.30, 49.92, 42.75, 39.82, 33.19; HRMS (ESI) m/z [M + H]^+^ calcd for C_16_H_23_N_4_O_2_^+^: 303.1816, found: 303.1816.

N′-[5-(4-Aminophenyl)-furan-2-yl-methyl]-N,N′-dimeth-ylethylenediamine Hydrochloride **(1c)**

General procedure F. Yellow solid (0.25 g, 95%); m.p. 60.2–61.1°C; ^1^H NMR (400 MHz, D_2_O) δ 7.76 (d, *J* = 8.7 Hz, 2H, Ar*H*), 7.33 (d, *J* = 8.7 Hz, 2H, Ar*H*), 6.84 (d, *J* = 3.5 Hz, 1H, Ar*H*), 6.77 (d, *J* = 3.5 Hz, 1H, Ar*H*), 4.47 (s, 2H, C*H*_*2*_), 3.54–3.46 (m, 2H, C*H*_*2*_), 3.46–3.39 (m, 2H, C*H*_*2*_), 2.85 (s, 3H, C*H*_*3*_), 2.67 (s, 3H, C*H*_*3*_); ^13^C NMR (101 MHz, D_2_O) δ 154.91, 142.33, 131.11, 125.52, 123.06, 118.03, 117.60, 107.61, 52.39, 49.92, 42.80, 39.86, 33.21; HRMS (ESI) m/z (M + H)^+^ calcd for C_15_H_22_N_3_O^+^: 260.1757, found: 260.1756.

N′-[5-(3-Aminophenyl)-furan-2-yl-methyl]-N,N′-dimethylethylenediamine Hydrochloride **(1d)**

General procedure F. Yellow solid (0.48 g, 95%); m.p. 64.2–65.2°C; ^1^H NMR (400 MHz, D_2_O) δ 7.71 (dd, *J* = 8.0, 1.4 Hz, 1H, Ar*H*), 7.59 (s, 1H, Ar*H*), 7.46 (t, *J* = 7.7 Hz, 1H, Ar*H*), 7.21 (ddt, *J* = 8.0, 2.2, 1.1 Hz, 1H, Ar*H*), 6.86 (d, *J* = 3.5 Hz, 1H, Ar*H*), 6.78 (d, *J* = 3.5 Hz, 1H, Ar*H*), 4.52 (s, 2H, C*H*_*2*_), 3.52–3.46 (m, 2H, C*H*_*2*_), 3.46–3.39 (m, 2H, C*H*_*2*_), 2.85 (s, 3H, C*H*_*3*_), 2.67 (s, 3H, C*H*_*3*_); ^13^C NMR (101 MHz, D_2_O) δ 154.52, 142.55, 131.33, 131.22, 130.76, 124.21, 122.31, 118.09, 117.63, 108.12, 52.39, 49.97, 42.80, 39.87, 33.24; HRMS (ESI) m/z (M + H)^+^ calcd for C_15_H_22_N_3_O^+^: 260.1757, found: 260.1756.

N′-[5-(4-N-Hydroxyamidino-phen-1-yl)-furan-2-yl-meth-yl]-N,N′-dimethylethylenediamine Hydrochloride **(1e)**

General procedure F. Yellow solid (0.51 g, 81%); m.p. 231.2–232.8°C; ^1^H NMR (400 MHz, D_2_O) δ 7.85 (d, *J* = 8.6 Hz, 2H, Ar*H*), 7.68 (d, *J* = 8.6 Hz, 2H, Ar*H*), 6.99 (d, *J* = 3.5 Hz, 1H, Ar*H*), 6.83 (d, *J* = 3.5 Hz, 1H, Ar*H*), 4.51 (s, 2H, C*H*_*2*_), 3.56–3.50 (m, 2H, C*H*_*2*_), 3.48–2.44 (m, 2H, C*H*_*2*_), 2.89 (s, 3H, C*H*_*3*_), 2.70 (s, 3H, C*H*_*3*_); ^13^C NMR (101 MHz, D_2_O) δ 161.16, 154.49, 143.14, 134.32, 128.29, 124.59, 124.09, 117.76, 109.33, 52.36, 50.02, 42.81, 39.90, 33.25; HRMS (ESI) m/z (M + H)^+^ calcd for C_16_H_23_N_4_O_2_^+^: 303.1816, found: 303.1805.

N′-[5-(3-Fluoro-4-N-hydroxyamidino-phen-1-yl)-furan-2-yl-methyl]-N,N′-dimethylethylenediamine Hydrochloride **(1f)**

General procedure F. Yellow solid (0.39 g, 91%); m.p. 216.8–218.6°C; ^1^H NMR (400 MHz, D_2_O) δ 7.66–7.57 (m, 3H, ArH), 7.00 (d, *J* = 3.6 Hz, 1H, ArH), 6.83 (d, *J* = 3.6 Hz, 1H, ArH), 4.49 (s, 2H, C*H*_*2*_), 3.54–3.50 (m, 2H, C*H*_*2*_), 3.48–3.42 (m, 2H, C*H*_*2*_), 2.87 (s, 3H, C*H*_*3*_), 2.70 (s, 3H, C*H*_*3*_); ^13^C NMR (101 MHz, D_2_O) δ 160.99, 158.47, 156.49, 153.30, 143.45, 136.20, 136.11, 130.44, 120.34, 117.80, 111.95, 111.83, 111.71, 110.24, 52.22, 50.02, 42.76, 39.82, 33.22; HRMS (ESI) m/z (M + H)^+^ calcd for C_16_H_22_FN_4_O_2_^+^: 321.1721, found: 321.1715.

N′-[5-(3-N-Hydroxyamidino-phen-1-yl)-furan-2-yl-methyl]-N,N′-dimethylethylenediamine Hydrochloride **(1g)**

General procedure F. White solid (0.41 g, 69%); m.p. 60.8–63.2°C; ^1^H NMR (400 MHz, D_2_O) δ 7.96–7.88 (m, 2H, Ar*H*), 7.55–7.50 (m, 2H, Ar*H*), 6.87 (d, *J* = 3.5 Hz, 1H, Ar*H*), 6.77 (d, *J* = 3.5 Hz, 1H, Ar*H*), 4.45 (s, 2H, C*H*_2_), 3.52–3.44 (m, 2H, C*H*_2_), 3.43–3.39 (m, 2H, C*H*_2_), 2.83 (s, 3H, C*H*_3_), 2.66 (s, 3H, C*H*_3_); ^13^C NMR (101 MHz, D_2_O) δ 161.20, 154.40, 142.61, 130.61, 130.09, 128.95, 127.05, 125.62, 122.98, 117.71, 108.22, 52.41, 50.03, 42.85, 39.89, 33.29; HRMS (ESI) m/z (M + H)^+^ calcd for C_16_H_23_N_4_O_2_^+^: 303.1816, found: 303.1811.

N′-[5-(4-Amidino-phen-1-yl)-furan-2-yl-methyl]-N,N′-dimethylethylenediamine Hydrochloride **(1h)**

General procedure F. White solid (330 mg, 81%); m.p. 186.5–188.9°C; ^1^H NMR (400 MHz, D_2_O) δ 7.82 (d, *J* = 8.6 Hz, 2H, Ar*H*), 7.72 (d, *J* = 8.6 Hz, 2H, Ar*H*), 6.96 (d, *J* = 3.5 Hz, 1H, Ar*H*), 6.80 (d, *J* = 3.5 Hz, 1H, Ar*H*), 4.47 (s, 2H, C*H*_*2*_), 3.52–3.48 (m, 2H, C*H*_*2*_), 3.45–3.40 (m, 2H, C*H*_*2*_), 2.85 (s, 3H, C*H*_*3*_), 2.67 (s, 3H, C*H*_*3*_); ^13^C NMR (101 MHz, D_2_O) δ 166.14, 154.49, 143.31, 134.54, 128.50, 126.96, 124.45, 117.73, 109.44, 52.40, 50.06, 42.89, 39.97, 33.28; HRMS (ESI) m/z (M + H)^+^ calcd for C_16_H_23_N_4_O^+^: 287.1866, found: 287.1869.

N′-[5-(3-Fluoro-4-amidino-phen-1-yl)-furan-2-yl-methyl]-N,N′-dimethylethylenediamine Hydrochloride **(1i)**

General procedure F. Yellow solid (300 mg, 80%); m.p.156.5–159.2°C; ^1^H NMR (400 MHz, D_2_O) δ 7.70–7.55 (m, 3H, Ar*H*), 7.00 (d, *J* = 3.6 Hz, 1H, Ar*H*), 6.82 (d, *J* = 3.5 Hz, 1H, Ar*H*), 4.49 (s, 2H, C*H*_*2*_), 3.55–3.48 (m, 2H, C*H*_*2*_), 3.47–3.41 (m, 2H, C*H*_*2*_), 2.86 (s, 3H, C*H*_*3*_), 2.69 (s, 3H, C*H*_*3*_); ^13^C NMR (101 MHz, D_2_O) δ 162.07, (161.17, 158.65), (153.42, 153.39), 143.56, (136.31, 136.21), 130.54, (120.26, 120.23), 117.80, (115.13, 115.01), (111.98, 111.74), 110.27, 52.27, 50.05, 42.80, 39.87, 33.23; HRMS (ESI) m/z (M + H)^+^ calcd for C_16_H_22_FN_4_O^+^: 305.1772, found: 305.1773.

N′-[5-(3-Amidino-phen-1-yl)-furan-2-yl-methyl]-N,N′-dimethylethylenediamine Hydrochloride **(1j)**

General procedure F. Orange oil (310 mg, 85%); ^1^H NMR (400 MHz, D_2_O) δ 7.99 (td, *J* = 1.9, 0.7 Hz, 1H, Ar*H*), 7.96 (dt, *J* = 7.7, 1.5 Hz, 1H, Ar*H*), 7.61 (dt, *J* = 7.9, 1.5 Hz, 1H, Ar*H*), 7.55 (td, *J* = 7.8, 0.6 Hz, 1H, Ar*H*), 6.89 (d, *J* = 3.5 Hz, 1H, Ar*H*), 6.78 (d, *J* = 3.5 Hz, 1H, Ar*H*), 4.45 (s, 2H, C*H*_*2*_), 3.51–3.44 (m, 2H, C*H*_*2*_), 3.45–3.38 (m, 2H, C*H*_*2*_), 2.83 (s, 3H, C*H*_*3*_), 2.67 (s, 3H, C*H*_*3*_); ^13^C NMR (101 MHz, D_2_O) δ 166.51, 154.51, 142.54, 130.45, 129.91, 129.11, 128.68, 127.22, 123.14, 117.68, 108.12, 52.37, 49.96, 42.78, 39.82, 33.23; HRMS (ESI) m/z (M + H)^+^ calcd for C_16_H_23_N_4_O^+^: 287.1866, found: 287.1867.

N′-[5-(3-Amidino-4-chloro-phen-1-yl)-furan-2-yl-methyl]-N,N′-dimethylethylenediamine Hydrochloride **(1k)**

General procedure F. Yellow solid (290 mg, 81%); m.p. 223–225°C; ^1^H NMR (400 MHz, D_2_O) δ 7.90–7.85 (m, 2H, Ar*H*), 7.60 (d, *J* = 8.5 Hz, 1H, Ar*H*), 6.91 (d, *J* = 3.5 Hz, 1H, Ar*H*), 6.83 (d, *J* = 3.5 Hz, 1H, Ar*H*), 4.52 (s, 2H, C*H*_*2*_), 3.62–3.50 (m, 2H, C*H*_*2*_), 3.52–3.42 (m, 2H, C*H*_*2*_), 2.89 (s, 3H, C*H*_*3*_), 2.72 (s, 3H, C*H*_*3*_); ^13^C NMR (101 MHz, D_2_O) δ 164.65, 153.39, 143.92, 130.96, 130.16, 129.02, 128.78, 128.44, 124.70, 117.01, 108.36, 52.39, 50.10, 43.19, 39.95, 33.15; HRMS (ESI) m/z (M + H)^+^ calcd for C_16_H_22_ClN_4_O^+^: 321.1477, found: 321.1465.

N′-[5-(2-Amidino-5-pyridyl)furan-2-yl-methyl]-N,N′-dimethylethylenediamine Hydrochloride **(1l)**

General procedure F. Yellow solid (200 mg, 70%); m.p. 75.8–78.2°C; ^1^H NMR (400 MHz, D_2_O) δ 9.03 (dd, *J* = 2.2, 0.8 Hz, 1H, Ar*H*), 8.28 (dd, *J* = 8.4, 2.2 Hz, 1H, Ar*H*)), 8.04 (dd, *J* = 8.4, 0.8 Hz, 1H, Ar*H*), 7.14 (d, *J* = 3.6 Hz, 1H, Ar*H*), 6.88 (d, *J* = 3.6 Hz, 1H, Ar*H*), 4.53 (s, 2H, C*H*_*2*_), 3.54 (m, 2H, C*H*_*2*_), 3.51–3.44 (m, 2H, C*H*_*2*_), 2.89 (s, 3H, C*H*_*3*_), 2.72 (s, 3H, C*H*_*3*_); ^13^C NMR (101 MHz, D_2_O) δ162.11, 151.75, 145.44, 144.21, 142.06, 132.78, 129.78, 123.33, 117.75, 111.05, 52.16, 50.05, 42.77, 39.80, 33.17; HRMS (ESI) m/z (M + H)^+^ calcd for C_15_H_22_N_5_O^+^: 288.1824, found: 288.1811.

N-Methyl-N′-[5-(2-amidino-5-pyridyl)furan-2-yl-methyl]-N′-cyclopropylethylenediamine Hydrochloride **(1m)**

General procedure F. Yellow solid (90 mg, 80%); m.p. 95.9–98.3°C; ^1^H NMR (400 MHz, D_2_O) δ 8.96 (dd, *J* = 2.3, 0.8 Hz, 1H, Ar*H*), 8.21 (dd, *J* = 8.4, 2.2 Hz, 1H, Ar*H*), 7.98 (dd, *J* = 8.4, 0.9 Hz, 1H, Ar*H*), 7.08 (d, *J* = 3.6 Hz, 1H, Ar*H*), 6.80 (d, *J* = 3.5 Hz, 1H, Ar*H*), 4.52 (s, 2H, C*H*_2_), 3.56–3.50 (m, 2H, C*H*_2_), 3.49–3.42 (m, 2H, C*H*_2_), 2.74 (tt, *J* = 7.3, 4.0°Hz, 1H, C*H*), 2.66 (s, 3H, C*H*_3_), 0.86 (dd, *J* = 7.1, 1.8°Hz, 2H, C*H*_2_), 0.75 (dd, *J* = 4.3, 2.0°Hz, 2H, C*H*_2_); ^13^C NMR (101 MHz, D_2_O) δ 162.01, 151.40, 145.40, 144.79, 141.86, 132.68, 129.82, 123.32, 117.64, 111.25, 51.92, 49.96, 42.92, 37.45, 33.24, 4.60; HRMS (ESI) m/z (M + H)^+^ calcd for C_17_H_24_N_5_O^+^: 314.1975, found: 314.1966.

N′-[5-(2-Amidino-5-pyridyl)furan-2-yl-methyl]-piperazine Hydrochloride **(1n)**

General procedure F. Yellow solid (200 mg, 85%); m.p. 197.7–199.3°C; ^1^H NMR (400 MHz, D_2_O) δ 8.96 (dd, *J* = 2.2, 0.9 Hz, 1H, Ar*H*), 8.21 (dd, *J* = 8.4, 2.2 Hz, 1H, Ar*H*), 7.98 (dd, *J* = 8.4, 0.9 Hz, 1H, Ar*H*), 7.06 (d, *J* = 3.5 Hz, 1H, Ar*H*), 6.77 (d, *J* = 3.6 Hz, 1H, Ar*H*), 4.38 (s, 2H, C*H*_2_), 3.44 [s, 4H, (C*H*_2_)_2_], 3.43 [s, 4H, (C*H*_2_)_2_]; ^13^C NMR (101 MHz, D_2_O) δ 162.09, 151.60, 145.40, 144.38, 141.96, 132.71, 129.80, 123.32, 117.46, 111.05, 52.30, 47.83, 40.86, 40.20; HRMS (ESI) m/z (M + H)^+^ calcd for C_15_H_20_N_5_O^+^: 286.1662, found: 286.1659.

N′-[5-(2-Amidino-5-pyridyl)furan-2-yl-methyl]-N′-methylethylenediamine Hydrochloride **(1o)**

General procedure F. Yellow solid (200 mg, 85%); m.p. 223.4–224.8°C; ^1^H NMR (400 MHz, D_2_O) δ 9.01 (s, 1H, Ar*H*), 8.24 (d, *J* = 8.2 Hz, 1H, Ar*H*), 8.02 (d, *J* = 8.6 Hz, 1H, Ar*H*), 7.07 (s, 1H, Ar*H*), 6.53 (s, 1H, Ar*H*), 3.71 (s, 2H, C*H*_2_), 3.01 (m, 2H, C*H*_2_), 2.68 (m, 2H, C*H*_2_), 2.27 (s, 3H, C*H*_3_); ^13^C NMR (101 MHz, D_2_O) δ 153.11, 149.12, 144.90, 140.54, 131.77, 130.43, 123.25, 112.67, 110.92, 52.64, 52.46, 40.78, 36.45; HRMS (ESI) m/z (M + H)^+^ calcd for C_14_H_20_N_5_O^+^: 274.1662, found: 274.1671.

N′-[2-(4-Amidino-phen-1-yl)thiazol-4-yl-methyl]-N,N′-dimethylethylenediamine **(1p)**

General procedure F. Gray powder (200 mg, 85%); m.p. 97.6–98.5°C; ^1^H NMR (400 MHz, D_2_O) δ 8.07–8.02 (m, 2H, Ar*H*), 7.86 (s, 1H, Ar*H*), 7.82–7.77 (m, 2H, Ar*H*), 4.53 (s, 2H, C*H*_2_), 3.62–3.54 (m, 2H, C*H*_2_), 3.49 (m, 2H, C*H*_2_), 2.88 (s, 3H, C*H*_3_), 2.69 (s, 3H, C*H*_3_); ^13^C NMR (101 MHz, D_2_O) δ 168.72, 165.92, 144.37, 136.79, 129.53, 128.59, 127.14, 125.36, 54.63, 50.31, 42.69, 40.08, 33.13; HRMS (ESI) m/z (M + H)^+^ calcd for C_15_H_22_N_5_S^+^: 304.1590, found: 304.1600.

N′-[2-(2-Aminocarboximido-5-pyridyl)thiazol-4-yl-methyl]-N,N′-dimethylethylenediamine Hydrochloride **(1q)**

General procedure F. Yellow solid (90 mg, 82%); m.p. 188.7–190.2°C; ^1^H NMR (400 MHz, D_2_O) δ 9.23 (dd, *J* = 2.3, 1.1 Hz, 1H, Ar*H*), 8.54–8.49 (m, 1H, Ar*H*), 8.15 (dd, *J* = 8.3, 1.2 Hz, 1H, Ar*H*), 7.80 (s, 1H, Ar*H*), 4.14 (s, 2H, C*H*_2_), 3.34 (m, C*H*_2_), 3.15 (m, 2H, C*H*_2_), 2.70 (s, 3H, C*H*_3_), 2.55 (s, 3H, C*H*_3_); ^13^C NMR (101 MHz, D_2_O) δ 164.53, 161.81, 147.43, 144.24, 135.94, 132.44, 123.40, 55.16, 51.00, 44.43, 40.51, 32.88; HRMS (ESI) m/z (M + H)^+^ calcd for C_14_H_21_N_6_S^+^: 305.1543, found: 305.1546.

N′-[2-(2-Aminocarboximido-5-pyridyl)thiazol-4-yl-methyl]-N′-methylethylenediamine Hydrochloride **(1r)**

General procedure F. Yellow solid (120 mg, 90%); m.p. 252–254°C; ^1^H NMR (400 MHz, D_2_O) δ 9.21 (dd, *J* = 2.3, 0.8 Hz, 1H, Ar*H*), 8.50 (dd, *J* = 8.4, 2.2 Hz, 1H, Ar*H*), 8.11 (dd, *J* = 8.3, 0.8 Hz, 1H, Ar*H*), 7.96 (s, 1H, Ar*H*), 4.58 (s, 2H, C*H*_2_), 3.57 (m, 2H, C*H*_2_), 3.48–3.43 (m, 2H, C*H*_2_), 2.91 (s, 3H, C*H*_3_); ^13^C NMR (101 MHz, D_2_O) δ 165.48, 160.87,147.71, 145.37, 144.76, 136.32, 132.48, 126.07, 123.58, 54.76, 51.80, 40.25, 34.03; HRMS (ESI) m/z (M + H)^+^ calcd for C_13_H_19_N_6_S^+^: 291.1386, found: 291.1380.

### Radioactive Methylation Assay and Selectivity Assay

The enzyme inhibitory activities and selectivity were measured by the radioisotope assay and AlphaLISA assay in ShangHai Chempartner Co., Ltd., according to the standard protocol. The radioactive methylation assay (Sun et al., 2019) was performed in a 1× assay buffer (modified tris buffer) system containing enzyme (PRMT1), peptide, and [^3^H]-SAM solution and compounds on the assay plate. After 250 nL of compound solutions were added to the assay plate, 15 μl of PRMT1 enzyme solution or ×1 assay buffer for negative control was transferred to each well of prepared compound stock plates and the whole system (the final concentration of PRMT1 was 0.5 nM or 2 nM in the system) was incubated for 15 min at room temperature. Then, 10 μl of peptide and (^3^H)-SAM mixed solution were added to each well to start the reaction (the final concentration of (^3^H)-SAM was 0.25 μM in the system) and the reaction was incubated for 60 min at room temperature. Afterward, the reaction was stopped with the addition of 5 μl cold SAM solution to each well. Then, 25 μl volume per well was transferred to a Flashplate from the assay plate and incubated for a minimum of 1 h at room temperature. Finally, the Flashplate was washed with dH_2_O and 0.1% Tween-20 three times and then the reading plate in Microbeta using the program ^3^H-Flashplate. The data were analyzed in GraphPad Prism 5 to obtain IC_50_ values.

The selectivity of **1r** (WCJ-394) against other type I PRMTs, including PRMT3, PRMT4, PRMT6, and PRMT8, was assessed using the AlphaLISA assay. In the AlphaLISA assay, firstly both the enzyme and substrate solution were prepared in a ×1 assay buffer, while compound **1r** was transferred to the assay plate by Echo in a final concentration of 1% DMSO. Next, 5 μl of enzyme solution was transferred to the assay plate and incubated at room temperature for 15 min. Then, 5 μl of substrate solution was added to each well to start the reaction. After incubation at room temperature for 30 min (PRMT4,6) and 60 min (PRMT3,8), 15 μl of acceptor and donor bead mix solution were added to the assay plate and incubated at room temperature for 60 min with subdued light. The signal was collected with Envision or EnSpire with Alpha mode. The data were analyzed in GraphPad Prism 5 to obtain IC_50_ values.

### Cell Lines and Cell Culture

The human lung epithelial cell line A549 was obtained from the American Type Culture Collection (ATCC, Manassas, VA, United States). Mink lung epithelial cells (MLECs) stably transfected with a TGF-β-responsive plasminogen activator inhibitor-1 promoter-luciferase construct were a kind gift from Dr. D Rifkin. The cells were cultured in Dulbecco’s modified Eagle’s medium (DMEM) with 10% fetal bovine serum (FBS), plus 1% penicillin and streptomycin (for A549 cells) or 200 μg/ml G418 (for MLEC) in a 37°C humidified environment containing 5% CO_2_.

### Cell Viability and Toxicity Assay

Cells were seeded into a 96-well plate; after 12 h of attachment, the cells were treated with different concentrations (0–1,600 μΜ) of WCJ-394 for 48 h. Cell viability was assessed by a Cell Counting Kit-8 (CCK-8; MCE) according to the manufacturer’s instructions. Briefly, after treatment, the CCK-8 solution was added to the culture medium and incubated at 37°C for 1 h. Cell viability was assessed by measuring the absorbance at 450 nm. In the subsequent luciferase assays, the effects of WCJ-394 on the cell viability were examined in parallel to exclude the compound-induced cell death.

### *In Vitro* Screening for the Inhibition of TGF-β Signaling

The ability of WCJ-394 to inhibit TGF-β signaling was assayed using MLEC stably transfected with a TGF-β-responsive plasminogen activator inhibitor-1 promoter-luciferase construct (Abe et al., 1994). Briefly, cells were seeded into a 96-well plate at a density of 1 × 10^4^ cells per well in DMEM with 10% FBS and incubated at 37°C for 6 h to adhere to the surface. The medium was changed into DMEM with 0.1% FBS. The cells were treated with different concentrations (0–100 μΜ) of WCJ-394 for 30 min before 0.05 ng/μl TGF-β1 (Peprotech) was added to the cells and then incubated for another 18 h. The final concentration of DMSO should be maintained at 0.1%. After incubation, the medium was removed and the cells were rinsed with PBS to remove any residual compound. One hundred microliters of the Steady-Glo^®^ Luciferase Assay reagent (Promega) was added to each well, and the cells were lysed in the dark for 10 min on a horizontal shaker. The luciferase activity was measured in a luminometer. The effect of WCJ-394 on MLEC cell viability was assessed by CCK-8 simultaneously.

### Western Blotting

A549 cells in the logarithmic growth phase were seeded into a 6-well cell culture plate at a density of 3 × 10^5^ cells per well. After an overnight incubation at 37°C in a 5% CO_2_ incubator, the medium was changed into the fresh DMEM with 0.1% FBS and the cells were incubated for another 24 h. The cells were treated with different concentrations (5–20 μΜ) of WCJ-394 for 2 h, with or without following the treatment of 5 ng/ml TGF-β1, and then incubated for another 48 h to measure the expression of EMT-associated proteins *via* the Western blotting assay. Additionally, A549 cells were treated with different concentrations of WCJ-394 (5–20 μΜ) to determine the expression of the PRMT 1-related protein.

A549 cells were lysed in RIPA buffer, which contains protease and phosphatase inhibitors, and then harvested in Eppendorf tubes for centrifugation (14,000 rpm for 10 min at 4°C) to remove cell debris. The supernatants were transferred into new Eppendorf tubes, and the protein concentration was quantified with a BCA kit (Thermo Fisher). The protein samples were separated by 10% SDS-PAGE and transferred onto polyvinylidene difluoride (PVDF) membranes. The membranes were blocked for 1 h at room temperature in 5% BSA and then incubated with the corresponding primary antibodies overnight at 4°C and then incubated with secondary antibodies for another 1 h at room temperature. After being washed with TBST buffer three times, the PVDF membranes were visualized with ECL reagents (Millipore) and the Western blot results were further analyzed by ImageJ software (National Institutes of Health).

### Scratch Wound Healing Assay

A549 cells in the logarithmic growth phase were seeded into 6-well plates at a density of 6 × 10^5^ cell per well and incubated at 37°C in a 5% CO_2_ incubator till reaching 100% confluence as a monolayer. The monolayer cell was scratched with a new 10 μl pipette tip across the center of the well, and the detached cells were gently rinsed with PBS buffer. The wells were refilled with a fresh DMEM medium that contains 0.1% FBS and the corresponding concentration of WCJ-394 to make the final concentration of 0, 5, 10, and 20 μΜ. After pretreatment with or without WCJ-394 for 2 h, TGF-β1 was added into the wells to a final concentration of 5 ng/ml and the cells were then incubated at 37°C in a 5% CO_2_ incubator for another 48 h. The scratch was photographed through a microscope at ×100 magnification at 0, 24, and 48 h. Then, the scratch width was quantified by ImageJ software and the rate of wound closure was calculated with the final values expressed as a migration percentage of the scratch width relative to 0 h.

### Transwell Migration Assay

A549 cells in the logarithmic growth phase were resuspended with a serum-free DMEM medium and seeded into the 8-μm transwell filter inserts at a density of 8 × 10^3^ cells per well; meanwhile, 600 μl medium containing 10% FBS was added into the lower chambers of the 24-well cell culture plate. The corresponding concentration of WCJ-394 was added into both the upper and lower chambers to reach a final concentration of 0, 5, 10, and 20 μΜ and incubated for 2 h. After the pretreatment of WCJ-394, the TGF-β1 was added as a chemoattractant into the lower chambers to a final concentration of 5 ng/ml and incubated for an additional 24 h at 37°C. After incubation, the cells in the upper chambers were carefully removed with a cotton swab, while the cells that had traversed to the bottom side of the membrane were fixed with 4% paraformaldehyde for 15 min and stained with 0.1% crystal violet for 20 min. The chambers were washed with PBS buffer to remove extra crystal violet. Three fields of view were randomly taken and photographed from the central and surrounding parts of each membrane under a microscope at ×100 magnification, and cell number was calculated by ImageJ software.

### Statistical Analysis

All the quantitative data are shown as mean ± SD from at least three independent experiments. Data were analyzed by using GraphPad Prism 8.0. Comparison of means of multiple groups was performed by a one-way analysis of variance (ANOVA). *p*-value below 0.05 was considered to be statistically significant.

### Molecular Docking

In the current study, the newly resolved crystal structure (PDB ID 6NT2) (Abe et al., 1994) of human PRMT1 complexed with GSK3368715 (a PRMT1 inhibitor) was downloaded from the Protein Data Bank. GOLD (Jones et al., 1997) (v5.2; Genetic Optimisation for Ligand Docking) was used for the docking study by referring the binding site of GSK3368715. Each compound was docked for 10 times, starting each time from a different random population of ligand orientations and using the default automatic genetic algorithm parameter settings. All torsion angles in each compound were allowed to rotate freely.

## Conclusion

In summary, based on our previous work, we designed and synthesized 15 2,5-substituted furan derivatives and 3 2,4-substituted thiazole derivatives. Among them, 10 compounds (**1h–k**, **1l,** and **1n–r**) showed strong inhibitory effects on PRMT1. Compound **1r** (WCJ-394) was identified as the most potent PRMT1 inhibitor (IC_50_ = 1.21 ± 0.11 μM) in the current study. Further experiments showed that WCJ-394 significantly downregulated the expressions of ADMA (the direct product of type I PRMTs) without affecting SDMA (the product of type II PRMTs) in A549 cells. The downregulation on the expression of DDAH indicated that WCJ-394 influenced the PRMT1–ADMA pathway *via* the inhibition on PRMT1. Moreover, WCJ-394 inhibited the TGF-β signaling pathway, which led to a downregulation on the expression of mesenchymal markers, so that the TGF-β-induced EMT was inhibited and the migration of A549 cells was prevented. The discovery of WCJ-394 proves to be very important for the understanding of the PRMT1 function in TGF-β signaling and is a potential lead compound for future PRMT1-related drug design.

## Data Availability

The original contributions presented in the study are included in the article/Supplementary Material; further inquiries can be directed to the corresponding authors.

## Abbreviations

ADMA, asymmetrical dimethylargine; CCK-8, cell counting kit-8; DDAH, dimethylarginine dimethylaminohydrolases; DMEM, Dulbecco’s modified Eagle’s medium; EMT, epithelial–mesenchymal transition; FBS, fetal bovine serum; MLECs, mink lung epithelial cells; MMA, monomethylarginine; MMPs, metalloproteinases; PRMT1, protein arginine methyltransferase 1; PVDF, polyvinylidene difluoride; SAH, S-adenosyl-L-homocysteine; SAM, S-adenosyl-L-methionine; SAR, structure–activity relationship; SDMA, symmetrical dimethylarginine; STAB, sodium triacetoxyborohydride; TGF-β, transforming growth factor β; α-SMA, alpha-smooth muscle actin.
